# Antiaromaticity in molecular assemblies and materials

**DOI:** 10.1039/d4sc05318d

**Published:** 2024-10-24

**Authors:** Roy Lavendomme, Masahiro Yamashina

**Affiliations:** a Laboratoire de Chimie Organique, Université libre de Bruxelles (ULB) Avenue F. D. Roosevelt 50, CP160/06 B-1050 Brussels Belgium roy.lavendomme@ulb.be; b Laboratoire de Résonance Magnétique Nucléaire Haute Résolution, Université libre de Bruxelles (ULB) Avenue F. D. Roosevelt 50, CP160/08 B-1050 Brussels Belgium; c Department of Chemistry, School of Science, Institute of Science Tokyo 2-12-1 Ookayama Meguro-ku Tokyo 152-8551 Japan yamashina.m.7af1@m.isct.ac.jp

## Abstract

Antiaromatic rings are infamously unstable and difficult to work with but they possess unusual electronic properties that make them interesting for fundamental and applied research. This perspective presents reports on discrete or polymeric assemblies made from antiaromatic building blocks, bound by either covalent linkages or supramolecular interactions. Compared to polymeric materials, discrete assemblies are more commonly studied, but most efforts have been devoted to their preparation and fundamental property studies, whereas applications are scarcely suggested. Future research in the field should focus on developing applications that benefit from the specific properties of antiaromatic rings. On the other hand, the few reports on antiaromatic-based materials hint at a promising future for this class of materials in organic electronics. To guide non-experts, different antiaromatic compounds are evaluated for their suitability as building blocks for larger assemblies.

## Abstract 2

Antiaromatic rings can be assembled into discrete or polymeric assemblies to explore emerging properties. This perspective compares antiaromatic rings as potential building blocks and covers reported antiaromatic ring assemblies.

## Graphical abstract

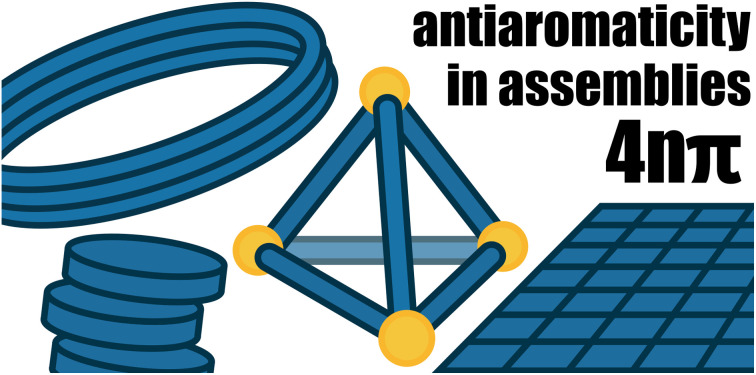

## 1. Introduction: antiaromaticity's attractiveness for molecular assemblies and materials

**Fig. 1:**
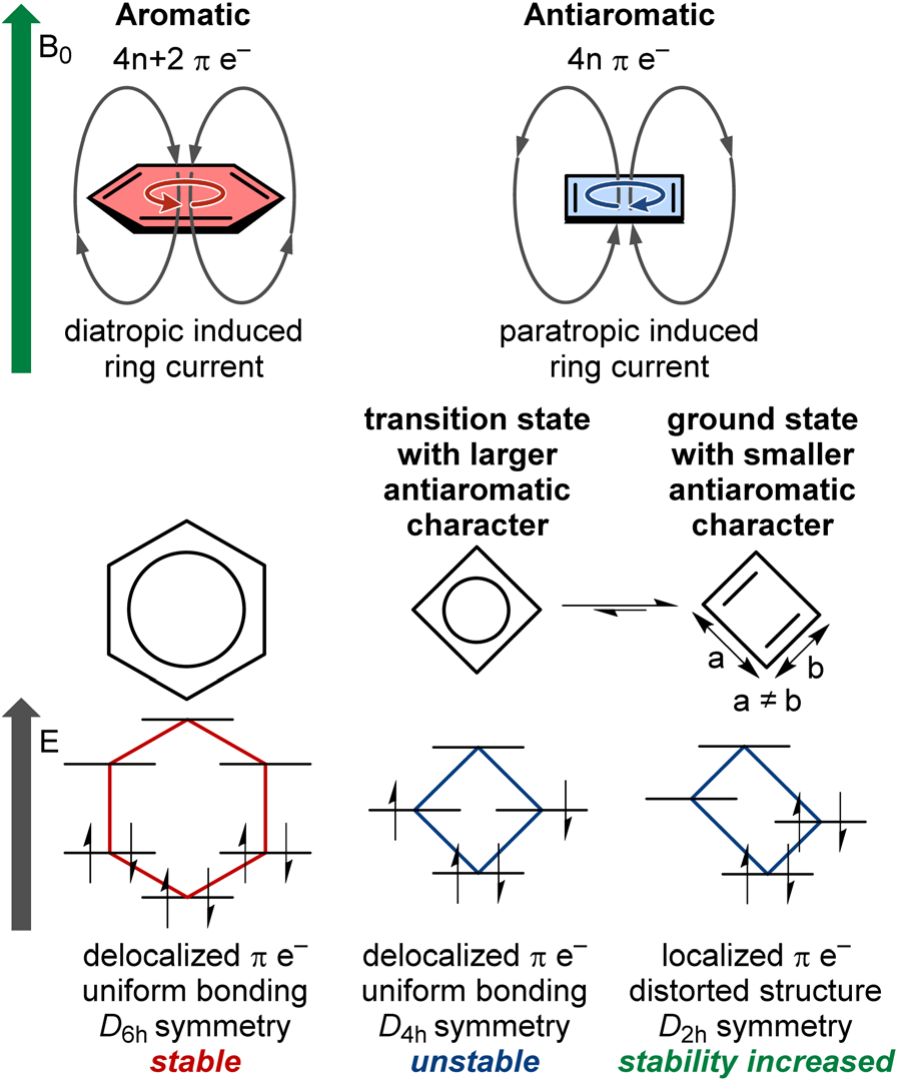
Key features differentiating aromatic and antiaromatic compounds exemplified by a benzene ring and a cyclobutadiene ring. *B*_0_ denotes the direction of an external magnetic field inducing ring current in the (anti)aromatic compounds.

Aromaticity is a fundamental concept in chemistry. In molecular structures in a singlet ground state, aromaticity arises when 4*n* + 2 electrons are delocalized in a conjugated π-system within planar rings, following Hückel's rule, among other descriptors.^1^ Aromatic rings exhibit increased stability, known as resonance energy, compared to the stabilization provided by conjugation alone. Aromatic molecules form a major class of compounds widely used in modern chemistry. They possess a range of interesting properties, such as electrical conductivity and diatropic ring current (Fig. 1). In contrast, antiaromaticity is characterized by a delocalized conjugated π-system of 4*n* electrons in planar rings, against Hückel's rule.^2^ Small antiaromatic rings with highly symmetric idealized structures may undergo structural distortion to increase stability *via* the Jahn–Teller effect.^3^ For instance, cyclobutadiene is more stable in the distorted *D*_2h_ rectangular form compared to the *D*_4h_-symmetric structure, which possesses a highly reactive diradical character (Fig. 1). This distortion tends to reduce π-electron delocalization and overall antiaromatic character (*e.g.*, cyclobutadiene and pentalene that remain planar and antiaromatic), or completely disrupt delocalization, leading to non-aromatic cycles (*e.g.*, cyclooctatetraene that adopts a non-planar saddle shape). Accordingly, in such cases, the antiaromatic character is mainly concentrated in highly symmetric transition states. For planar conjugated rings in their lowest triplet state, the (anti)aromaticity is better described by Baird's rule, where 4*n* π-electrons denote aromaticity and 4*n* + 2 π-electrons denote antiaromaticity.^4–6^ It should be noted that there is no consensus on the exact definition of (anti)aromaticity or its best descriptors.^7–9^ The present perspective does not aim to discuss or establish such definitions. Instead, we focus on singlet ground state antiaromatic compounds, using the commonly accepted Hückel's rule and ring current concepts described above.

Antiaromatic rings typically exhibit low stability and have remained an oddity in fundamental research for decades. However, some antiaromatic ring structures with sufficient stability to be isolated, studied, and used for applications have been sporadically reported.^10^ These reports have shown that antiaromatic compounds possess specific interesting properties such as high conductivity,^11^ multi-redox behavior,^12^ biradical character,^13,14^ closed-shell paramagnetism,^15^ and a paratropic ring current that strengthens external magnetic fields (Fig. 1).^16^ These properties have been used to tune the reactivity of molecules by pushing a reaction from an unstable antiaromatic intermediate to a stable aromatic product,^17,18^ or to develop electric circuit-based devices,^19^ for instance.

One research direction in the field of antiaromatic compounds is assembling or connecting them with other compounds (antiaromatic or not) through supramolecular or covalent interactions, and to determine what properties may emerge in the newly formed superstructures. In this perspective, we present diverse antiaromatic ring motifs and assess their respective benefits for developing antiaromatic-based assemblies. We then provide an overview of reported antiaromatic-based discrete or polymeric assemblies. Finally, we explore the potential of antiaromatic-based assemblies for practical applications.

## 2. Choosing suitable antiaromatic building blocks

When aiming to build supramolecular or covalent assemblies based on antiaromatic rings, the following points should be considered when selecting antiaromatic building blocks:

(i) They should be easily functionalizable to introduce the desired chemical groups that will allow for assembly.

(ii) They should possess geometrical features compatible with the desired assembly, such as topicity, symmetry, planarity, and bulkiness. For instance, assembling a supramolecular cube of octahedral symmetry would require *D*_4h_-symmetric (planar square) molecules, although closely related cuboid assemblies could also be formed from *C*_4v_-symmetric (bent square) or *D*_2h_-symmetric (planar rectangle) building blocks.

(iii) They should be sufficiently stable to survive the assembly conditions and allow the assembly to have a lifespan suitable for the desired applications. This point is especially crucial for antiaromatic compounds, which notoriously display low stability.

(iv) To make the best use of antiaromatic building blocks in applications, they should possess a strong antiaromatic character. Indeed, akin to aromatic rings that can exhibit varying intensities of aromaticity depending on the degree of π-electron delocalization within the cycle, antiaromatic rings also display a range of antiaromatic intensities.

The antiaromatic intensity can be estimated from the strength of the paratropic ring current generated in the antiaromatic ring when exposed to a magnetic field. A convenient, although imperfect, method to estimate this paratropic ring current intensity is the calculation of nucleus-independent chemical shifts (NICSs) using density functional theory (DFT).^20–22^ NICS values may vary with the chosen DFT functional, leading to significant differences,^23^ but good agreement is often observed for small molecules. Positive NICS values at the center of the ring (NICS(0)) or along the central perpendicular axis (*e.g.*, NICS(1) for 1 Å distance from the center) indicate a paratropic ring current and antiaromaticity, whereas negative values indicate a diatropic ring current and aromaticity. The most commonly reported NICS type is the isotropic NICS_iso_ which is the average of the three diagonal elements of the shielding tensor (*xx*, *yy*, and *zz*); however (anti)aromaticity in planar rings is better described by the *zz* shielding tensor component, NICS_*zz*_.^24^ Accordingly, small antiaromatic compounds can be assigned relative antiaromaticity intensities or strengths (Table 1). Note that NICS values are not directly comparable across rings of different sizes and a correction parameter should be applied. Other complementary or alternative descriptors of (anti)aromaticity may also prove to be useful for characterizing antiaromatic rings, such as anisotropy of the induced current density (ACID),^25^ gauge-including magnetically induced currents (GIMICs),^26^ aromatic ring current shieldings (ARCS),^27^ and topological resonance energy (TRE).^28,29^

**Table 1:** Properties of selected small molecules containing an antiaromatic ring

Antiaromatic compound family	NICS(0)_iso_ (ppm)^a^	NICS(1)_*zz*_ (ppm)^a^	HOMO–LUMO gap (eV)^a^^,^^b^	Main rotation axis	Stability^c^	Antiaromatic area relative to π-surface^d^
Cyclobutadiene 1 (*D*_2h_)	25.7	59.8	3.70	*C*_4_	Low	100%
Pentalene 2 (*C*_2h_)	24.7	62.3	2.62	*C*_2_	Low	100%
Dibenzo[*a*,*e*]pentalene 3	11.0	26.9	3.14	*C*_2_	High	41%
Dihydrodiborinine 4 (R = Me)^e^	10.3	16.7	4.05	*C*_2_	Low	100%
Dihydroboranthrene 5 (R = Me)^e^	10.2	16.5/16.7^g^	4.19	*C*_2_	Medium	37%
Dihydropyrazine 6 (R = Me, *D*_2h_)^e^	19.8	53.9	3.54	*C*_2_	Low	100%
Dihydrophenazine 7 (R = Me)^e^	5.3	17.6/15.9^g^	4.44	*C*_2_	High	33%
Tetraaza[8]circulene 8 (R = Me)^e^	8.4	23.2	3.24	*C*_4_	High	22%
Tetraoxa[8]circulene 9	8.4	23.1	3.68	*C*_4_	High	22%
Tetrathia[8]circulene 10	6.8	18.5	3.65	*C*_4_	High	22%
Tetraselena[8]circulene 11	7.3	18.8	3.41	*C*_4_	High	21%
M-norcorrole 12 (M = Ni, R = Mes)^e^^,^^f^	43.5	123.6/102.0^g^	1.51	*C*_2_	High	100%
	43.7	132.8/106.4^g^				
Isophlorin 13 (R = Me)^e^	19.9	60.1/59.1^g^	1.69	*C*_4_	High	100%
Tetraoxaisophlorin 14a (R = Me)^e^	25.4	72.1/70.9^g^	1.64	*C*_4_	High	100%
Tetraoxaisophlorin 14b (R = CF_3_)^e^	5.7	21.7/22.0^g^	2.25	*C*_4_	High	100%
Tetraoxaisophlorin 14c (R = Ph)^e^	22.1	63.6/63.8^g^	1.59	*C*_4_	High	100%
Tetraoxaisophlorin 14d (R = C_6_F_5_)^e^	21.2	60.9/61.0^g^	1.63	*C*_4_	High	100%
Tetraoxaisophlorin 14e (R = *p*-NO_2_-Ph)^e^	17.4	51.6/51.8^g^	1.54	*C*_4_	High	100%
Tetrathiaisophlorin 15 (R = Me)^e^	16.7	52.9/41.7^g^	1.76	*C*_4_	High	100%
Tetraselenaisophlorin 16 (R = Me)^e^	−1.3	10.8/7.9^g^	3.08	*C*_4_	High	100%

a Calculations were performed at the B3LYP/6-31G(d) level of theory.

b HOMO–LUMO gaps should only be compared between compounds with identical conjugation patterns (*e.g.*14a and 14b).

c Broad evaluation of stability for each family of compounds, based on the ease of synthesis and modification reported in the literature, may vary with substituents.

d Rings of the DFT-optimized structures were partitioned into triangles from their centroid to calculate their surface area.

e The R groups based on structures in Fig. 2 were selected to perform geometry optimization and NICS calculations; Me as a small inert group; Mes as the most common side moiety on M-norcorrole.

f NICS values are given for the center of both the 6- and 5-membered rings including the metal of M-norcorrole.

g The induced magnetic field is anisotropic on either asymmetric side of non-planar structures, thus NICS(1)_*zz*_ and NICS(−1)_*zz*_ values are given for both directions perpendicular to the ring.

A selection of small molecules containing an antiaromatic ring in their neutral state is presented in Fig. 2 and Table 1. The NICS values in Table 1 were calculated for DFT-optimized structures at the B3LYP/6-31G(d) level of theory using the Gaussian 16 program.^30^ For small antiaromatic rings such as cyclobutadiene^31^1 and pentalene^32,33^2, with 4 and 8 conjugated π-electrons, respectively, strong antiaromaticity is associated with low stability. Accordingly, these compounds are difficult to functionalize and are not suitable building blocks. One strategy to increase the stability of these small antiaromatic rings is to combine them with aromatic rings in conjugated polycyclic compounds (*e.g.*, benzoannulated dibenzopentalenes^34,35^ and hetero[8]circulenes,^36,37^ see Table 1). This approach decreases the antiaromatic strength (NICS(1)_*zz*_ = 62.3 ppm for pentalene 2*vs.* 26.9 ppm for dibenzo[*a*,*e*]pentalene 3) while increasing stability by favorably delocalizing the electrons over the aromatic circuit instead of the antiaromatic circuit. Antiaromatic rings stabilized in this manner have been extensively used as building blocks, as detailed below.

**Fig. 2:**
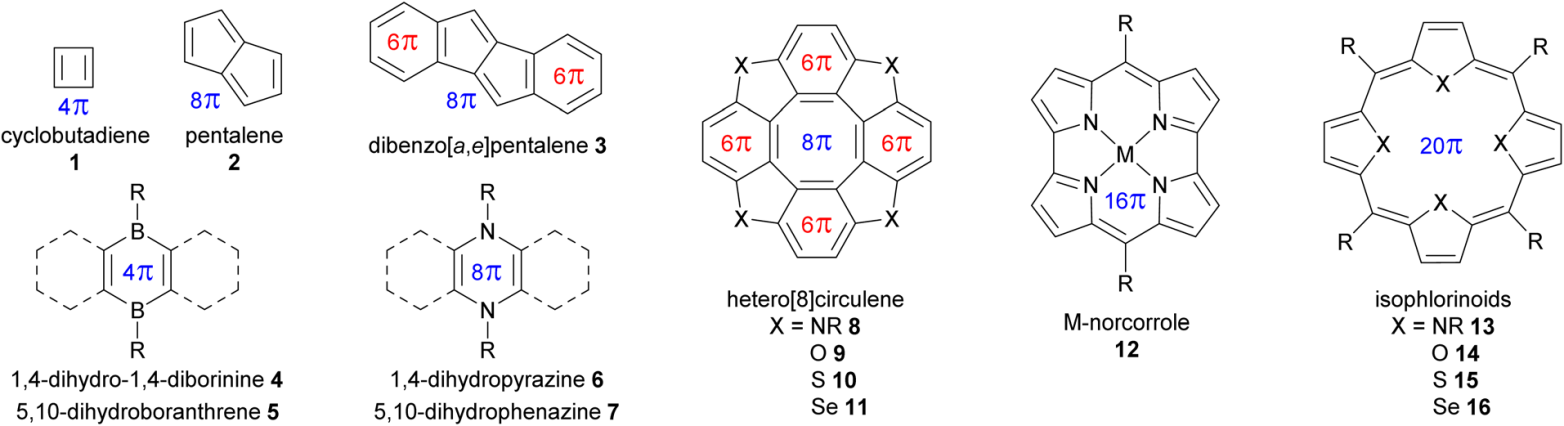
Selection of small molecules containing an antiaromatic ring. π-electrons are shown for antiaromatic rings (4*n* in blue) and aromatic rings (4*n* + 2 in red).

Another strategy to increase the stability of antiaromatic rings is to attach electron-withdrawing groups that will deplete the electron density of the antiaromatic ring. For example, tetraoxaisophlorin derivatives 14a–e exhibit decreasing antiaromatic strength (according to NICS calculations) and larger HOMO–LUMO gaps with increasing electron-withdrawing ability of *meso*-substituents (*e.g.*, –CH_3_14a*vs.* –CF_3_14b, and phenyl 14c*vs.* pentafluorophenyl 14d*vs. p*-nitrophenyl 14e, see Table 1). Interestingly, this difference is less pronounced in the case of pentafluorophenyl *meso*-substituents governed by inductive electron-withdrawing effects compared to *p*-nitrophenyl ones governed by stronger mesomeric effects. In accordance with this stabilization trend, Reddy and Anand showed that the electron-depleted antiaromatic ring of 14d was more resistant to oxidation into the aromatic dication, compared to 14c.^38^

Borole,^39^ dihydrodiborinine 4, and dihydroboranthrene 5 derivatives contain small antiaromatic rings with 4 π-conjugated electrons, but the nature of the boron atom with its Lewis acid character imparts high reactivity toward Lewis bases, allowing them to form Lewis adducts or undergo nucleophilic substitution. As a result, they are not the most convenient building blocks for antiaromatic assemblies, although their reactivity might be useful in switching devices. Nonetheless, fused rings with these antiaromatic organoboron rings have exhibited interesting properties, such as stabilization of radicals^40^ and high quantum yields.^41^

The derivatives of dihydropyrazine^42^6 and dihydrophenazine^43,44^7 have been extensively studied for their specific redox and photophysical properties, which allow for tuning their shape and reactivity.^45,46^ Despite their sufficient stability for modification and study, there have been no reports of large assemblies using these antiaromatic motifs as building blocks. This may be due to the predominance of aromatic rings fused to the antiaromatic core, limiting the potential use of the latter in larger assemblies. It is noteworthy that the dihydropyrazine motif occurs in nature and is a key structural element in redox-active biological molecules such as flavins^47^ and some luciferins.^48^

Strongly luminescent nonacene derivatives containing both dihydroborinine 4 and dihydropyrazine 6 cores to tune the charge separation properties have recently been reported, showcasing the useful electronic and photophysical properties of antiaromatic compounds.^49–51^

Cyclooctatetraene (*i.e.*, [8]annulene) containing 8 π-electrons naturally adopts a saddle shape, preventing conjugation and thus avoiding the instability caused by antiaromaticity. In hetero[8]circulenes 8–11, the planarity and conjugation of the central cyclooctatetraene are imposed by the lateral fused aromatic rings, creating a central antiaromatic core.^36^ Hetero[8]circulenes are excellent building blocks for assemblies due to their main *C*_4_ symmetry axis, high stability, and interesting photophysical properties,^52^ as demonstrated by the numerous examples detailed below. However, their main limitation is the relatively weak antiaromaticity of the cyclooctatetraene core, imparted by the fused aromatic rings wherein the ring current is mainly concentrated.^53^ Examples of mixed-heteroatom hetero[8]circulenes provide additional degrees of freedom for tuning reactivity and properties.^54^ It should be noted, however, that the large selenium atoms in tetraselena[8]circulenes 11 prevent perfect planarity, leading to a certain degree of saddle shape. Therefore, the planar tetraaza-, oxa-, and thia[8]circulenes 8–10 are preferable candidates for use as building blocks in assemblies.

With the increasing size of π-conjugated macrocycles such as annulenes^55^ and trannulenes,^56^ antiaromatic molecules may exhibit a significant increase in stability compared to smaller cycles such as cyclobutadiene. Some large antiaromatic heteromacrocycles, such as M-norcorroles^57^12 and isophlorinoids^58^13–16, with 16 and 20 conjugated π-electrons, respectively (see Table 1), show strong antiaromaticity and sufficient stability for functionalization, despite the absence of fused aromatic rings in contrast to hetero[8]circulenes 8–11, for instance. This may be due to the absence of a highly symmetric degenerate open-shell ground state (unlike *D*_4h_ cyclobutadiene in Fig. 1). Indeed, the different bonds in the antiaromatic macrocycle are inherently differentiated, promoting localized π-bonding without needing Jahn–Teller distortion, thus reducing the antiaromatic character to a more stable domain. Such antiaromatic rings hold great potential as building blocks for larger assemblies with emerging properties, owing to their strong antiaromatic character, as detailed in the following sections. Ni-norcorrole benefits from an optimized large-scale synthesis method,^57^ along with the possibility of attaching functional groups suitable for assembly directly to the norcorrole moiety,^59–62^ although this remains synthetically challenging. However, M-norcorroles present some limitations as potential building blocks, such as their low degree of symmetry, with a main *C*_2_ axis. In comparison, isophlorinoids appear to be better candidates for assemblies, offering short—but often low-yielding—synthesis pathways and geometrical features (*e.g.*, a *C*_4_ symmetry axis) identical to those of porphyrins, which are extensively used in assemblies like covalent organic cages,^63^ metal–organic cages,^64^ covalent organic frameworks (COFs),^65^ and metal–organic frameworks (MOFs).^66^ Their strong antiaromatic character gives rise to closed-shell paramagnetism.^67^ Interestingly, antiaromatic isophlorins with pyrrole (NH) moieties spontaneously oxidize to the well-known aromatic porphyrins when exposed to air. However, it is possible to substitute the pyrrole units to replace the central H atoms with boron,^68^ carbon,^69^ or silicon,^70^ for instance, stabilizing the isophlorin against oxidation to preserve antiaromaticity. Similar to tetraselena[8]circulenes 11, the large selenium atoms in tetraselenaisophlorin 16 preclude the planarity of the macrocycle, thus decreasing the degree of conjugation and antiaromaticity compared to other isophlorinoids, as demonstrated by the NICS values in Table 1. Like hetero[8]circulenes, isophlorinoids can also be synthesized with mixed heteroatoms, providing more degrees of freedom for tuning their properties.^38,71,72^

Many other antiaromatic structures are not detailed here, including some that are suitable for making assembled antiaromatic structures, such as indenofluorene,^73,74^ indacene,^75^ indolocarbazole,^76^ [6]cyclo-*para*-phenylmethine,^77^ and dihydrodiazapyrene.^78^ Some of these antiaromatic molecules don't meet the criteria for good building blocks, such as expanded azacoronene^79,80^ and corrole^81^ derivatives that possess a low degree of symmetry, or expanded porphyrinoids^82–84^ and furan-acetylene macrocycles,^85^ which are impractical from a synthetic standpoint.

## 3. Discrete molecular assemblies and architectures with antiaromatic character

Numerous discrete molecular assemblies and architectures (*e.g.*, rings and cages) have been reported in the literature, and their properties in terms of structure, host–guest systems, and reactivity have been extensively studied.^86,87^ Compared to aromatic-based systems, those based on antiaromatic motifs are far less reported owing to the difficulty in functionalizing their scaffolds. Due to their antiaromatic character, there are essentially two ways to obtain antiaromatic-based architectures: (i) functionalizing native antiaromatic building blocks to assemble them, and (ii) converting aromatic-based architectures to antiaromatic ones *via* redox reactions. The former involves using antiaromatic molecules that are relatively stable under ambient conditions. The latter involves controlling the number of π-electrons in pre-synthesized molecular architectures through redox reactions. In this section, we introduce recent achievements in constructing antiaromatic molecule-based architectures through these two approaches.

### 3.1. Antiaromaticity from natively antiaromatic building blocks

There are two common approaches in chemistry to assemble building blocks into discrete architectures: assembly *via* covalent bonding or non-covalent interactions.

The first approach, covalent bonding, led to the synthesis of [*n*]cyclo-*para*-phenylenes ([*n*]CPPs)^88^ and related cylindrical molecules,^89^ in which aromatic panels are circularly connected through C–C covalent bonds, constituting one of the most important classes of nanocarbons.

**Fig. 3:**
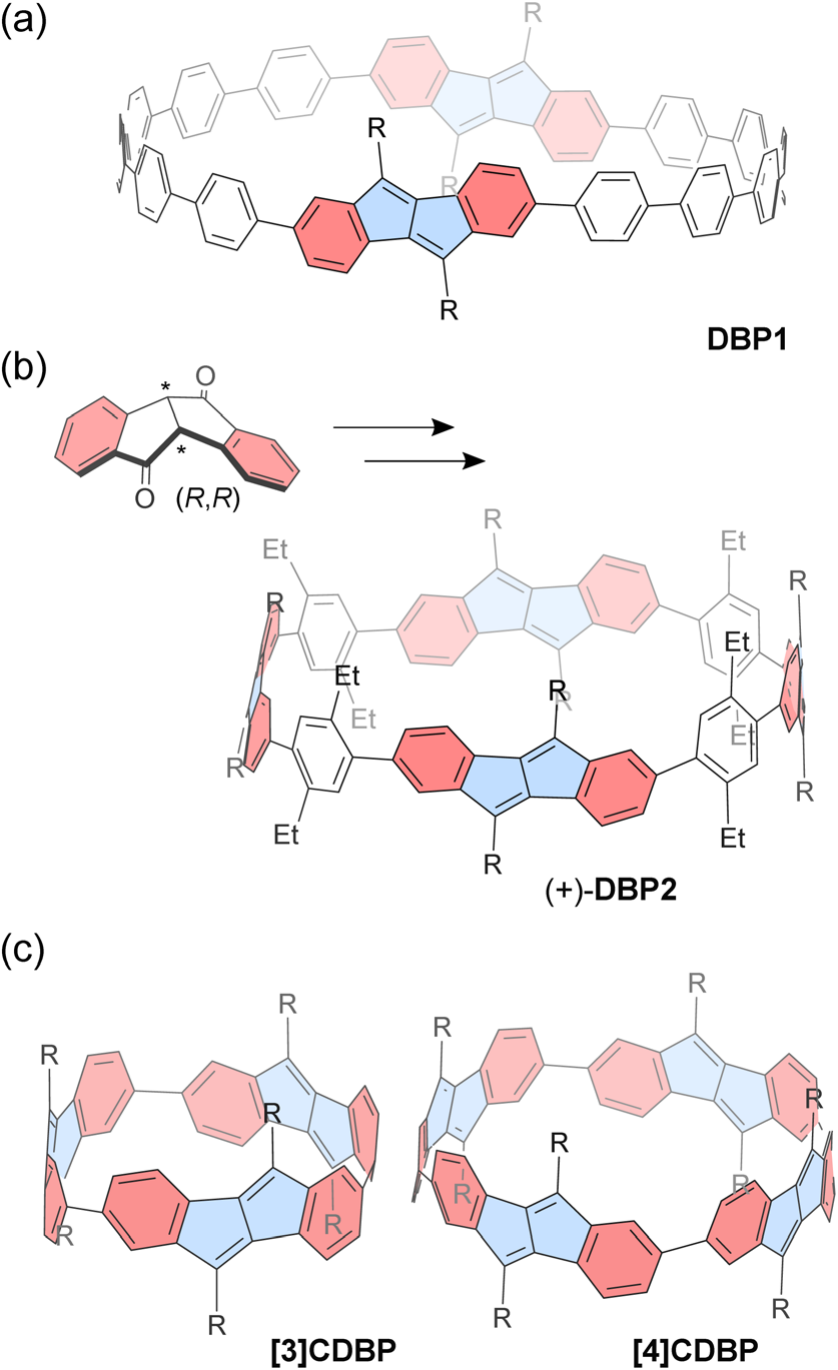
Chemical structures of dibenzo[*a*,*e*]pentalene-based macrocycles (a) DBP1, (b) DBP2, (c) [3]CDBP and [4]CDBP. Different subunits are colored as follows: blue for antiaromatic pentalene and red for aromatic benzene.

Esser and co-workers successfully reported a series of CPP macrocycles containing antiaromatic dibenzo[*a*,*e*]pentalene (DBP) units (Fig. 3).^90^ The antiaromatic DBP can be derived from corresponding bent-shaped diketone compounds. For instance, a [12]CPP containing two DBP units (DBP1) is synthesized *via* Suzuki–Miyaura coupling and Yamamoto coupling reactions, followed by oxidative aromatization. According to DFT calculations, the optimized structure of DBP1 has a diameter of *ca.* 2.1 nm and a strain energy of 36.5 kcal mol^−1^, similar to that of [16]CPP. Owing to its relatively large diameter, the incorporated DBP shows a small bent angle of 36.7° and a torsion angle of 36° between the neighboring phenyl rings. Incorporating DBP units provides the CPP macrocycle with altered redox potential and absorption spectra. In particular, the antiaromatic DBP units within the CPP show a slightly reduced antiaromatic character as a consequence of π-extension through sexiphenyl linkers, according to NICS calculations.

The chiral DBP-based π-conjugated nanorings (±)-DBP2 (Fig. 3) were synthesized under stereoselective control from enantiomerically pure bent-shaped diketone precursors by the same group.^91^ The enantiopure diketones are conveniently obtained *via* sulfoximine-mediated optical resolution. Owing to the bent-shaped diketone scaffold, the subsequent homo- and cross-coupling reactions towards the formation of macrocyclic precursor proceed with good yields. Finally, a Grignard reaction followed by dehydrative aromatization produces the corresponding enantiopure (+)-DBP2 and (−)-DBP2 nanorings, respectively. The resulting nanorings do not racemize, even at 110 °C as confirmed with electronic circular dichroism spectra and molecular dynamics (MD) simulations. The antiaromaticity of the DBP units is slightly reduced owing to the π-conjugation with phenyl rings, and this is reflected in the macrocycle's ambipolar electrochemical properties, which allow for reversible reduction and two quasi-reversible oxidations. It should be noted that DBP2 has a relatively large diameter of 2.5 nm, allowing it to accommodate up to two C_60_ molecules with binding constants *K*_11_ = (5.4 ± 0.7) × 10^3^ M^−1^ and *K*_12_ = (1.1 ± 1.0) × 10^2^ M^−1^ in toluene-*d*_8_. The proton NMR signals of the nanoring's substituents show downfield shifts as C_60_ concentration increases, owing to the deshielding effect from C_60_.

In contrast to the abovementioned nanorings with phenylene spacers, [*n*]cyclodibenzopentalenes ([*n*]CDBPs), in which some DBP units are directly connected, have been recently reported.^92^ A platinum-mediated macrocyclization of dibenzopentalene diboronic esters produced two types of [*n*]CDBPs (*n* = 3, 4) (Fig. 3). The cyclic trimer [3]CDBP and tetramer [4]CDBP have high strain energies of 80 and 64 kcal mol^−1^, respectively. According to NMR spectroscopy and DFT calculations, each ring exists as a single diastereomer (*D*_n_ symmetry) in solution. X-ray crystallography revealed that the [4]CDBP molecules align to form columnar structures in the solid state. Strong π-conjugation around the ring leads to high HOMO energies and small band gaps, along with a slight decrease in local antiaromaticity within the DBP units. The relatively large diameter of 1.3 nm for [4]CDBP is suitable for binding either fullerenes C_60_ or C_70_ in a 1 : 1 complex, exhibiting slow exchange on the NMR chemical shift timescale with C_60_ and fast exchange with C_70_. The corresponding activation energies for guest encapsulation are higher than those of [10]CPP.

As shown in the above macrocycles, even for aromatic systems, synthesizing discrete architectures often requires multiple synthetic steps and competes with the formation of undesired oligomers, leading to low yields. To alleviate this limitation, the second approach for preparing discrete architectures, which involves the assembly of building blocks *via* non-covalent interactions, has proven to be a powerful tool. Notably, self-assembly based on multiple non-covalent interactions is one of the most powerful methods for quantitatively constructing high-ordered molecular assemblies from simple monomeric building blocks.^87^ This approach works due to well-designed monomeric molecules that spontaneously assemble into thermodynamically-favored molecular architectures.

**Fig. 4:**
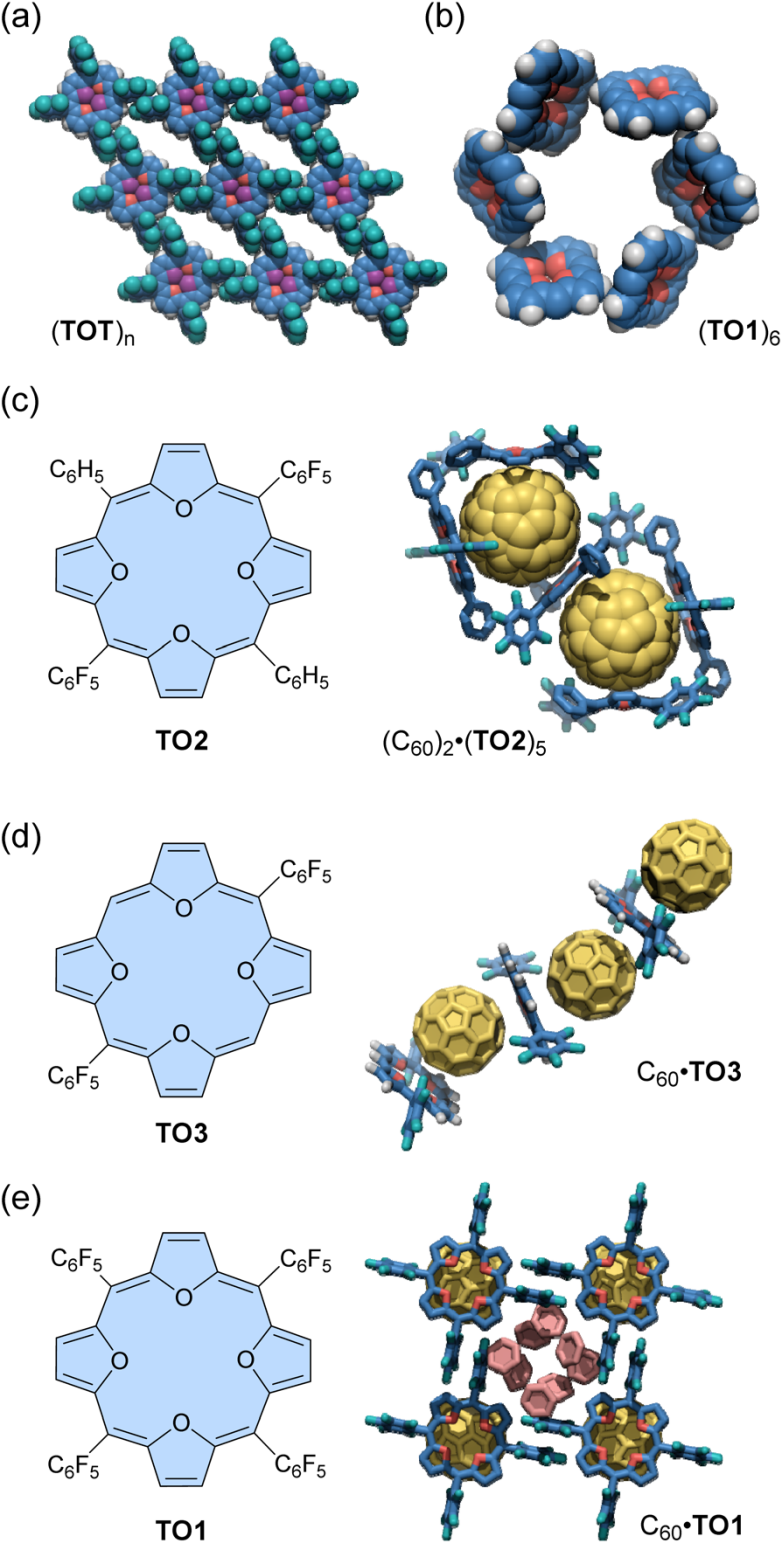
Crystalline-induced isophlorin-based assemblies: (a) (TOT)_*n*_ and (b) (TO1)_6_ (*meso*-C_6_F_5_ groups are omitted for clarity), and host–guest complexes, (c) (C_60_)·(TO2)_5_ (hydrogen atoms are omitted for clarity), (d) C_60_·TO3, and (e) C_60_·TO1 (hydrogen atoms are omitted for clarity). The color scheme for the crystal structures: H (white), C (blue, yellow, and salmon), O (red), F (teal), and S (purple).

An early example of the exploration of antiaromatic molecule-based assemblies was reported by Anand and co-workers in 2008.^93^ Tetraoxaisophlorin, a relatively stable antiaromatic porphyrinoid, has a large π-surface akin to a porphyrin. Anand's team synthesized two types of tetrakis(pentafluorophenyl)isophlorins with either a furan core (*i.e.*, tetraoxaisophlorin, TO1) or a furan/thiophene core (TOT), respectively (Fig. 4). As mentioned in the previous section, these isophlorin derivatives exhibit strong antiaromaticity due to their 20 π-electron systems. X-ray analysis revealed that TO1 and TOT form supramolecular arrangements through self-complementary C–H⋯F–C hydrogen bonding in the solid state. For the isophlorin TOT, the *para*-fluorine of all four *meso*-pentafluorophenyl rings and the four β-hydrogens of the two thiophenes participate in C–H⋯F–C interactions (2.68 Å, 171°). These C–H⋯F–C interactions around each macrocycle lead to a well-defined assembly of a two-dimensional rhomboid grid (Fig. 4a). Some solvent molecules are found within the voids. Similar intermolecular C–H⋯F–C interactions are also observed for tetraoxaisophlorin TO1. In addition, an intermolecular C–H⋯π interaction (2.74 Å, 133°) is observed between the β-hydrogen of a furan from one molecule and the furan π-surface in another molecule. As a consequence of self-complementary C–H⋯π interactions between six molecules, tetraoxaisophlorin TO1 forms a cyclic hexamer (TO1)_6_ in the solid state (Fig. 4b). Furthermore, three similar F⋯F interactions (2.91 Å) are observed in this assembly. A combination of these non-covalent interactions supposedly acts as the driving force for the assembly of TO1 in the form of a cyclic hexamer (TO1)_6_ in the crystal packing.

Anand's group also demonstrated the formation of antiaromatic molecule-based host–guest complexes through co-crystallization.^94^ Diphenyl dipentafluorophenyl-substituted tetraoxaisophlorin (TO2) co-crystallizes with fullerene C_60_ from a toluene/acetone solution to yield black-colored crystals (Fig. 4c). In these crystals, a fullerene molecule is surrounded by three molecules of tetraoxaisophlorin TO2 in a triangular arrangement, forming the (C_60_)_2_·(TO2)_5_ host–guest complex. In this complex, one TO2 is intercalated between the two encapsulated fullerenes, preventing any fullerene–fullerene contact. The tetraoxaisophlorin TO2 surface is found to be extremely close to the C_60_ π-surface, with a C–C distance comparable to that observed between C_60_ and a free-base aromatic porphyrin (<2.76 Å). The *ortho*-F atoms of pentafluorophenyl groups have short contacts with C_60_ (*ca.* 3.1 Å). In contrast to the (C_60_)_2_·(TO2)_5_ assembly, the co-crystallization of TO2 with excess fullerene yields another assembly (TO2)·(C_60_)_2_, where the macrocycle is sandwiched between two fullerene units.

The other tetraoxaisophlorin derivatives, dipentafluorophenyl-substituted TO3 and tetraphenyl-substituted TO1, form 1 : 1 complexes with C_60_ in an alternating tetraoxaisophlorin–C_60_ arrangement. The C_60_ complex of TO3 forms a zig-zag assembly (Fig. 4d), whereas that of TO1 forms a linear-chain (Fig. 4e). Among these complexes, the π-surface of tetraoxaisophlorins interacts with C_60_ through strong π–π interactions (distance of *ca.* 2.6 Å).

The tetraoxaisophlorin–C_60_ complexation was also evidenced in the solution state. Solutions of tetraoxaisophlorins TO2 and TO3 show a distinct color change from green to brown upon the addition of C_60_, suggesting the formation of the complex. A 1 : 1 solution of TO2 and C_60_ in toluene-*d*_8_ displays a 0.03 ppm upfield shift for the β hydrogens of furan and a 0.03 ppm downfield shift for the *meso* phenyl hydrogens. A 0.10 ppm shift is observed for the *ortho* fluorine atoms by ^19^F NMR spectroscopy. ^13^C NMR spectroscopy shows negligible signal shifts. A titration study revealed that TO2 and TO3 bind C_60_ to form a 1 : 1 complex in a chloroform/toluene solution with association constants of 9.91 × 10^2^ M^−1^ and 7.16 × 10^3^ M^−1^ for TO2·C_60_ and TO3·C_60_, respectively. According to thermodynamic parameters, the complexation between these tetraoxaisophlorins and C_60_ results from both enthalpically and entropically favored processes.

**Fig. 5:**
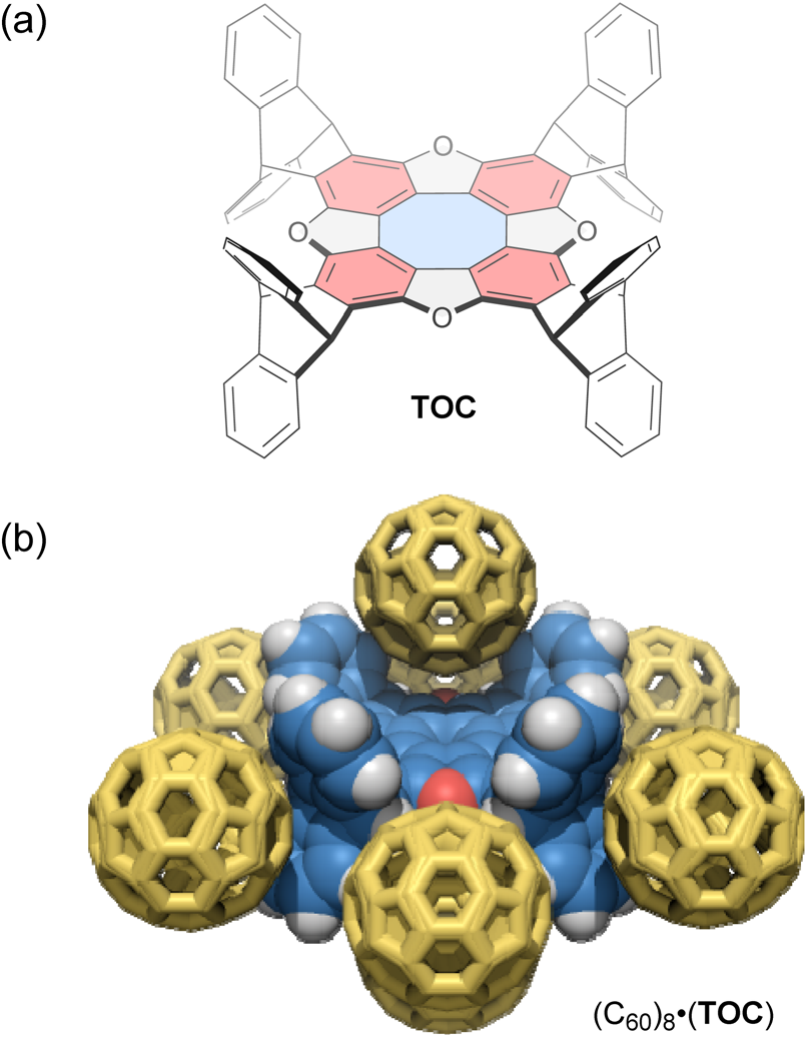
(a) Chemical structure of tetraoxa[8]circulene-based host TOC (blue: antiaromatic cyclooctatetraene; red: aromatic benzene) and (b) its host–guest complex with C_60_, (C_60_)_8_·(TOC). The color scheme for the crystal structure: H (white), C (blue and yellow), and O (red).

Tetraoxa[8]circulenes include a formally antiaromatic cyclooctatetraene core. Wuest and co-workers reported various “awkwardly” shaped tetraoxa[8]circulene derivatives with large concave electron-rich aromatic surfaces.^95^ These derivatives have a *D*_4h_-symmetric structure with aromatic rings protruding on both sides of the *σ*_h_ plane (Fig. 5a), resulting in improved solubility and synthesis efficiency compared to previously reported circulenes. One derivative, TOC, forms C_60_-encapsulated mixed crystals in a 1 : 2 host–guest stoichiometry *via* co-crystallization. In these crystals, each molecule of TOC interacts directly with a total of eight molecules of C_60_; two C_60_ molecules occupy offset positions above and below the tetraoxa[8]circulene core, while the remaining six molecules surround the outside of the core (Fig. 5b). Upon C_60_ inclusion, the cyclooctatetraene core of the host exhibits significantly altered C–C bond lengths compared to those in 1,2-dichlorobenzene and CHCl_3_ co-crystals, likely due to a gain or loss of aromaticity. In addition, in a CHCl_3_ solution, the characteristic fluorescence of the tetraoxa[8]circulene core is quenched upon adding 1,4-dinitrobenzene, but not by hydroquinone. This result shows the potential of tetraoxa[8]circulenes and related compounds as optical sensors for electron-deficient species.

**Fig. 6:**
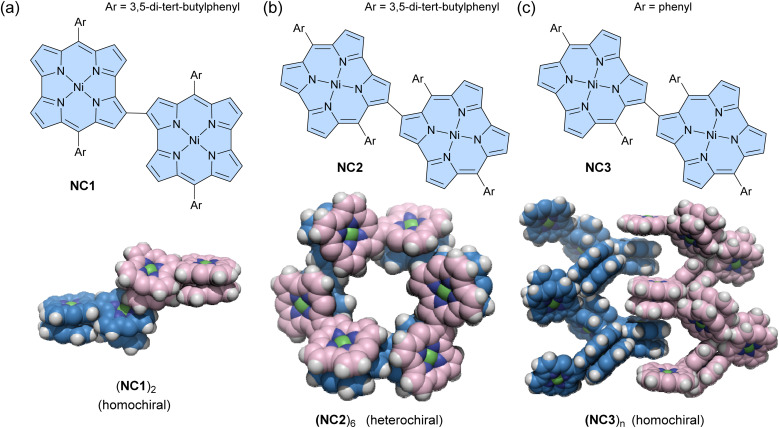
Chemical structures of norcorrole-based dimers and their assemblies in the solid state. (a) NC1 and (NC1)_2_, (b) NC2 and (NC2)_6_, and (c) NC3 and (NC3)_*n*_. The color scheme for the crystal structures: H (white), C (blue for (*R*)-isomers and pink for (*S*)-isomers), N (dark blue), and Ni (green). Ar groups are omitted for clarity.

Some antiaromatic molecules (*e.g.*, norcorrole and hexaphyrin) are known to adopt a face-to-face π-stacking arrangement, exhibiting stacked-ring aromaticity, due to effective intermolecular orbital interaction.^96–100^ Utilizing this property, Shinokubo and co-workers recently reported three types of norcorrole dimers that form various assemblies in both solution and crystalline states.^101^ A 2,2′-linked norocorrole dimer with *meso*-3,5-di-*tert*-butylphenyl groups NC1 assembles as a π-stacked dimer (NC1)_2_*via* face-to-face π-interactions between each norcorrole molecule in the solid state (Fig. 6a). In contrast, a 3,3′-linked norcorrole dimer with *meso*-3,5-di-*tert*-butylphenyl groups NC2 and a dimer with *meso*-phenyl groups NC3 form macrocyclic hexamers (NC2)_6_ and helical assemblies (NC3)_*n*_ in the solid state, respectively (Fig. 6b and c). Interestingly, racemic mixtures of norcorrole dimers (*R*- and *S*-) exhibit chiral self-sorting behavior depending on the linking position and *meso*-aryl substituents. Homochiral self-sorting occurs for (NC1)_2_ and (NC3)_*n*_, while heterochiral self-sorting occurs for (NC2)_6_. The π-stacked dimer (NC1)_2_ also forms in a toluene solution with an association constant of (3.6 ± 1.7) × 10^5^ M^−1^ at 20 °C. After dimerization, the absorption spectrum shows a broad absorption band at *ca.* 860 nm, characteristic of face-to-face stacked norcorroles. In contrast, dimers NC2 and NC3 do not form (NC2)_2_ and (NC3)_*n*_ assemblies in solution. However, the ^1^H NMR spectrum of NC2 shows that the pyrrole signals are significantly downfield shifted and desymmetrized at −60 °C, suggesting the formation of some aggregates. This can be considered an example of solid-state self-assembly, demonstrating different assembly behaviors between solution and solid states.^102^

**Fig. 7:**
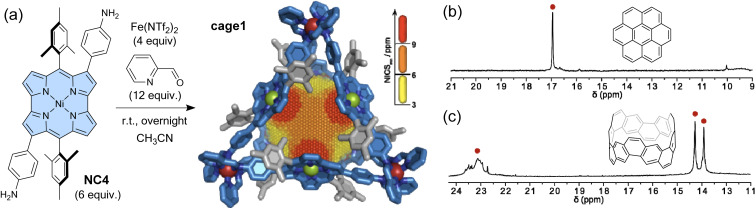
(a) Synthesis of a norcorrole-based supramolecular cage with an antiaromatic-walled nanospace from NC4. The X-ray crystal structure of cage1 in stick representation with a three-dimensional NICS grid, showing the magnetic deshielding experienced within the antiaromatic-walled nanospace. Partial ^1^H NMR spectra (500 MHz, CD_3_CN, 298 K) showing the encapsulated guest signals of (b) cage1·(coronene)_2_ and (c) cage1·(carbon nanobelt). The color scheme for the crystal structure: C (blue and grey), N (dark blue), Fe (red), and Ni (green). Hydrogen atoms are omitted for clarity.

As mentioned in Section 1, antiaromatic molecules produce an induced magnetic field, which is inverted compared to aromatic molecules, due to paratropic ring currents (Fig. 1). The induced magnetic field significantly affects the ^1^H NMR signals. In cavitands where antiaromatic molecules face the cavity (*i.e.* the guest recognition site), guest molecules should experience deshielding effects from the cavity walls. Nitschke and co-workers constructed a coordination-driven supramolecular cage based on norcorrole and demonstrated the intermolecular deshielding effect *via* molecular encapsulation in solution.^103^ Mixing 2,6-bis(3-aminophenyl)norcorrole (NC4), 2-pyridine carboxyaldehyde, and Fe(NTf)_2_ yielded an M_4_L_6_-type tetrahedral supramolecular cage cage1*via* subcomponent self-assembly (Fig. 7). NMR, MS, and X-ray analyses confirmed the formation of the cage structure with a mass of *ca.* 7000 Da. 2D and 3D NICS calculations revealed a magnetically deshielded nanospace where antiaromatic moieties surrounding the cavity reinforce each other. Indeed, the NICS value at the center of the cage, formed by 6 norcorrole units, is larger than the additive NICS values of the six independent units. The antiaromatic-walled nanocage encapsulates various polycyclic aromatic molecules (*e.g.*, coronene and carbon nanobelt) in acetonitrile. As expected, the ^1^H NMR signals of the encapsulated guests were observed at chemical shift values of up to 24 ppm, shifted by 16 ppm from that of the free guest, due to the combined deshielding effects of the surrounding antiaromatic rings. In particular, the degree of shift in the guest signals varies depending on the type of molecule, ranging from 3 to 16 ppm. This result indicates variability in the deshielding regions within the cavity. The deshielding effect diminishes towards the vertex areas, where aromatic units (*i.e.*, phenyl and pyridine moieties) are densely packed. This cage may thus be considered a type of NMR shift reagent and opens the way for further probing the effects of antiaromatic environments within a nanospace.

**Fig. 8:**
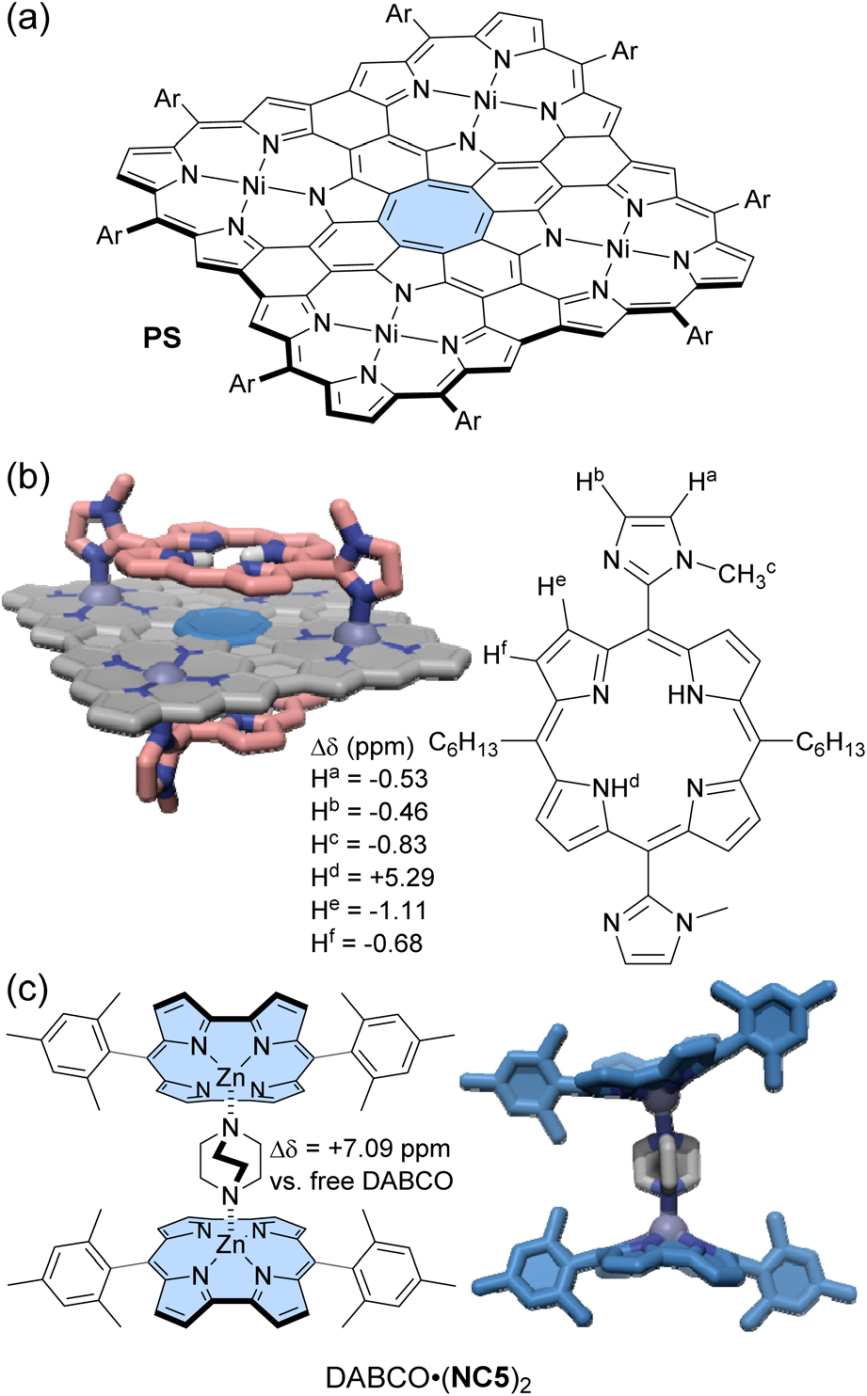
(a) Planar cyclooctatetraene incorporated porphyrin nanosheet PS and (b) its 1 : 2 host–guest complex with bisimidazolyl porphyrin guests whose NH located in front of the antiaromatic cyclooctatetraene experiences strong deshielding. (c) Chemical structure of DABCO·(NC5)_2_ and its X-ray crystal structure. The color scheme for the crystal structures: H (white), C (grey, pink, and blue), N (dark blue), and Zn (blue-grey). Non-polar hydrogen atoms and side chains are omitted for clarity.

Peek and co-workers synthesized a Zn(ii)-norcorrole NC5 as its pyridine complex (py·NC5) *via* metalation of free-base norcorrole.^104^ The strong deshielding effect of norcorrole caused the signals of the coordinated pyridine to appear at 13.95 and 9.38 ppm. Upon axial coordination, the norcorrole plane becomes significantly curved into a bowl-shaped structure. In the ^1^H NMR spectrum, the *o*-methyl groups of the mesityl substituents showed a difference of 2.36 ppm in the chemical shift between the convex and concave sides of NC5. Cyclic voltammetry measurements suggested that the curvature of the norcorrole plane weakened the overlap of π-orbitals, reducing the degree of antiaromaticity. When DABCO (1,4-diazabicyclo[2.2.2]octane), a bis-monodentate ligand, was added to py·NC5, either a 1 : 1 or 1 : 2 complex of NC5 and DABCO formed, depending on the DABCO molar ratio (Fig. 8c). In toluene-*d*_8,_ the ^1^H NMR signals of DABCO in the DABCO·(NC5)_2_ complex appear significantly downfield shifted at 9.52 ppm (Δ*δ* = 7.09 ppm), although those in the DABCO·NC5 complex appeared at 7.69 and 4.05 ppm (Δ*δ*_max_ = 5.26 ppm). This difference is due to the cooperative deshielding effect of the two antiaromatic norcorrole fragments within DABCO·(NC5)_2_. The 1 : 1 binding constant of DABCO to py·NC5 is approximately 3 orders of magnitude higher than that for an aromatic Zn(ii)-porphyrin, likely reflecting that the norcorrole's curvature better exposes the Zn(ii) for axial binding.

**Fig. 9:**
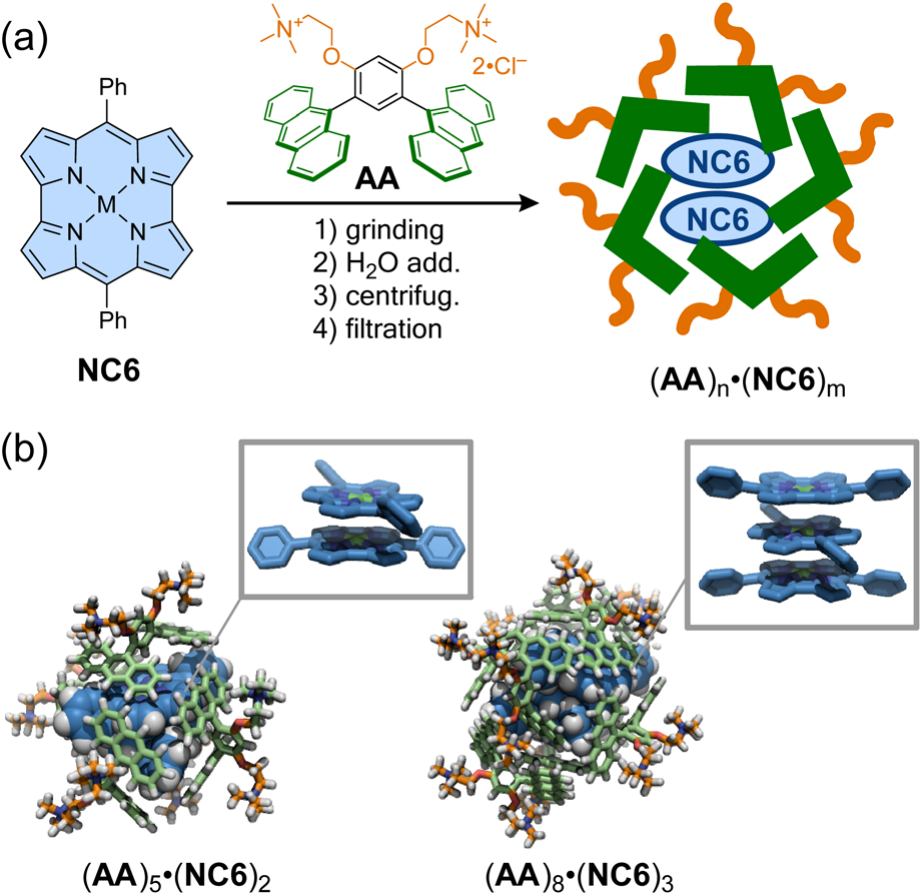
(a) Encapsulation of *meso*-phenyl Ni(ii)-norcorrole (NC6) within anthracene-based micellar capsules to form (AA)_*n*_·(NC6)_*m*_. (b) Optimized structures of (AA)_5_·(NC6)_2_ and (AA)_8_·(NC6)_3_ and their highlighted guest stacking. The color scheme for the models: H (white), C (blue, pale green, and orange), N (dark blue), and Ni (green).

As in the representative example of Cram,^105^ molecular encapsulation of an antiaromatic guest by a host molecule can stabilize and reveal the specific properties of the antiaromatic molecules. Shinokubo, Yoshizawa, Kim, and co-workers reported that a norcorrole exhibits stacked-ring aromaticity within an anthracene-based micellar host through molecular encapsulation. A nanocapsule composed of bent polyaromatic amphiphiles AA was employed to encapsulate some molecules of *meso*-phenyl Ni(ii)-norcorrole (NC6) in water *via* the hydrophobic effect (Fig. 9).^106^ Dynamic light scattering and ^1^H NMR analyses showed that the resulting micellar capsules (AA)_*n*_·(NC6)_*m*_ are *ca.* 2 nm in size, with host–guest ratios estimated to be 2.5 : 1. Within the capsules, the encapsulated norcorroles exhibited a broad absorption band in the near-infrared region, characteristic of norcorroles with close face-to-face stacking, resulting in stacked-ring aromaticity. The structure of (AA)_*n*_·(NC6)_*m*_ was simulated using DFT and molecular mechanics calculations, revealing that double- and triple-decker stacked norcorroles are wrapped with five or eight amphiphiles to form host–guest complexes (AA)_5_·(NC6)_2_ (2.4 nm) and (AA)_8_·(NC6)_3_ (2.7 nm). Interestingly, a *meso*-isopropyl norcorrole, which does not exhibit π-stacking even in concentrated solution or crystalline form, displayed π-stacking with stacked-ring aromaticity in the micellar capsule. This supramolecular approach thus provides a facile strategy for achieving π–π stacking of antiaromatic compounds without laborious synthesis.

### 3.2. Antiaromaticity originating from redox reactions

In contrast to the direct assembly of antiaromatic building blocks through covalent bonding or non-covalent interactions, a third approach involves the preliminary assembly of discrete architectures from aromatic building blocks, followed by the conversion of the aromatic units into antiaromatic units *via* redox reactions. The number of π-conjugated electrons in ring systems can be switched between 4*n* + 2 (aromatic) and 4*n* (antiaromatic) by redox reactions. Notably, when oxidation or reduction reactions are performed on macrocyclic π-conjugated molecules (*i.e.* [*n*]annulenes and [*n*]trannulenes), global aromaticity or antiaromaticity may arise, in which the ring current extends throughout the macrocycle.^55,107,108^ For example, the π-conjugated system of [8]cycloparaphenylene ([8]CPP), in which the benzene rings are connected at the *para* position, is localized on each benzene ring in the neutral state. However, in the dication ([8]CPP^2+^), which is chemically oxidized using NOSbF_6_ or SbCl_5_, the entire ring exhibits an in-plane conjugated π-system based on quinoid structures, achieving global aromaticity throughout the ring.^109^ Recently, the global aromaticity of dicationic [6]CPP^2+^ acting as a wheel in rotaxanes was evidenced by ^1^H NMR measurements, which showed that the proton signal of the central axle molecule is affected by a significantly greater shielding effect compared to the neutral form.^110^ These examples validate the use of redox reactions to impart global aromaticity to large macrocycles, but achieving global antiaromaticity in this manner may be more challenging since the notoriously less stable antiaromatic electronic configuration needs to be somehow stabilized in a redox-active environment, where it could favorably switch to another stable aromatic configuration. For instance, it is essentially difficult to synthesize a highly oxidized state (+4) in CPP exhibiting global antiaromaticity. In contrast, it has been reported that the reduction of [8]CPP with alkali metals yields [8]CPP^4−^, which has global antiaromaticity, but its properties in the solution state are not clear.^111,112^

**Fig. 10:**
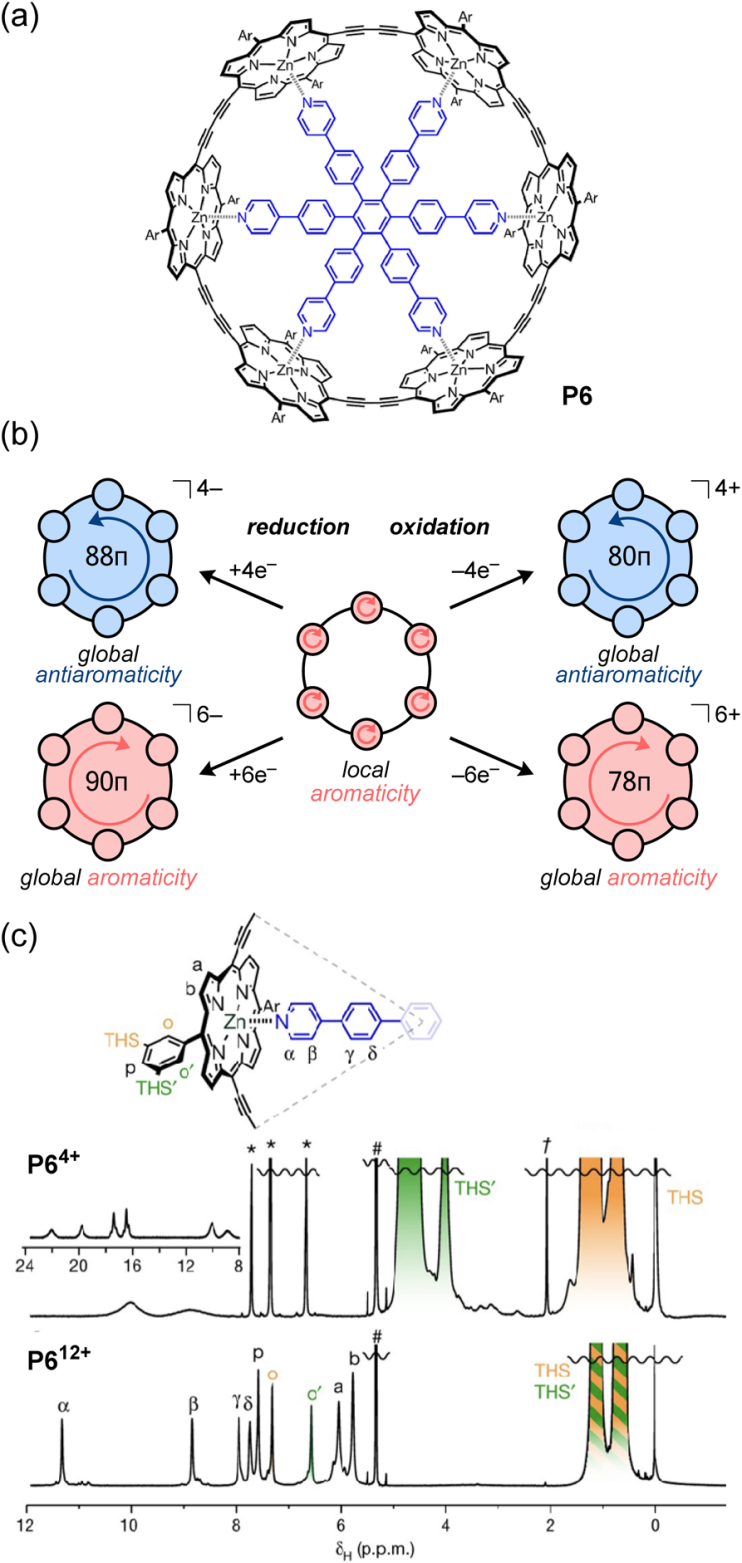
(a) Chemical structure of the porphyrin nanoring P6. (b) Expression of global aromaticity/antiaromaticity of P6 induced by redox reactions; the arrows indicate the direction of the ring current induced by a magnetic field perpendicular to the plane directed toward the observer. (c) ^1^H NMR spectra (500 MHz, CD_2_Cl_2_, 223 K) of P6^4+^ (top) and P6^12+^ (bottom).

In this context, Anderson and co-workers found that porphyrin nanorings exhibit global aromaticity or antiaromaticity depending on their oxidation state (Fig. 10).^113^ Their porphyrin nanoring P6 has 84 π-electrons and can attain the +4, +6, and +12 oxidation states *via* chemical oxidation with oxidants (*e.g.*, tris(2,4-dibromophenyl)aminium hexafluoroantimonate; DIBAHA_F_). In the case of neutral porphyrin nanoring P6, NICS calculations reveal that the aromatic shielding effect is localized above and below the plane of each porphyrin moiety, indicating no signs of global aromaticity or antiaromaticity. Indeed, ^1^H NMR measurements display no significant change in the chemical shift difference (Δ*δ* = *δ*_inner_ − *δ*_outer_) between the outer and inner trihexylsilyl (THS) group protons of the ring. In contrast, NICS calculations for P6^4+^ (80π) reveal positive NICS values (deshielding) inside the ring, and negative values (shielding) outside, which are characteristic of paratropicity and global antiaromaticity. From ^1^H NMR measurements, the paratropic ring current causes a large chemical shift difference (Δ*δ* = +3.45 ppm) between the THS protons inside and outside the ring. Due to the strong deshielding effect from the P6^4+^ ring, the ^1^H signal of the pyridyl group of the template appears around 23 ppm (Fig. 10c). On the other hand, P6^6+^ shows global aromaticity, as evidenced by the negative chemical shift difference (Δ*δ* = −0.70 ppm) for the THS protons and the pyridyl proton signals appearing around 5.5 ppm. In the P6^12+^ ring with the highest oxidation state, NICS calculations show that the ring exhibits local antiaromaticity at each porphyrin unit in the 16π dicationic oxidation state, but no global antiaromaticity. Accordingly, the THS proton signals inside and outside the ring appear almost equivalent. Although the antiaromatic porphyrin units deshield the pyridyl protons of the template (*δ* = 8–12 ppm), the degree of deshielding is weaker than that for the P6^4+^ ring with global antiaromaticity. It was originally expected that global antiaromaticity in porphyrin nanorings would require a central template to lock them in a cylindrical shape and prevent the out-of-plane conformational twisting of the porphyrin units, which could disrupt the destabilizing antiaromatic conjugation. However, the Anderson group later found that the positive charge generated upon oxidation effectively locks the nanoring into a cylindrical shape, even in the absence of a template, thus allowing global antiaromaticity in untemplated nanorings.^114^

The same group investigated the global aromaticity of nanorings by systematically varying the number of π-electrons by altering the number of –C≡C– units and porphyrins.^115^^1^H NMR analyses showed that the difference in chemical shifts between the THS groups inside and outside the ring decreases as the number of π-electrons increases. The smallest ring, with 68 π-electrons in its tetracationic state, showed the largest shifts in THS signals, and the signal of the pyridyl group of the template appeared at 36 ppm. The global aromaticity/antiaromaticity of these nanorings can be evaluated through ^19^F NMR measurements of fluorinated templates and ^1^H NMR. For the largest 12-porphyrin nanoring, with 168 π-electrons in the neutral state, the ^1^H signal of the template was significantly broadened. However, clear signal changes due to the different oxidation states were observed in the ^19^F NMR spectra. In contrast, when this nanoring is twisted into a figure-of-eight shape with two smaller templates, the two loops experience induced ring currents in opposite directions that cancel each other out; consequently the resulting NMR spectra showed no detectable ring currents. The NMR spectra thus showed almost no change in chemical shifts for the template signals, regardless of the oxidation state.

In contrast to these oxidation reactions, there are examples of global aromaticity/antiaromaticity derived from reduction reactions.^116^ The porphyrin ring P6 can be reduced to the P6^4−^ and P6^6−^ states (Fig. 10b). NICS calculations show that the tetraanion P6^4−^ is antiaromatic (88 π-electrons), while the hexaanion P6^6−^ is aromatic (90 π-electrons). The ^1^H NMR spectrum of P6^4−^, obtained by adding 4 equivalents of decamethylcobaltocene 
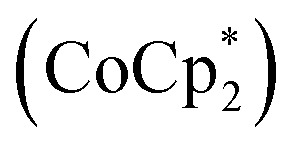
 to a P6 solution in THF-*d*_8_, showed a strong deshielding effect due to global antiaromaticity, with the template pyridyl signals appearing up to 18 ppm. The addition of excess 
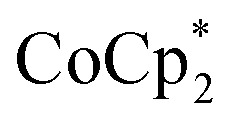
 to a solution of P6 in THF yielded a reddish-brown solution corresponding to P6^6−^. The ^1^H NMR spectrum revealed that P6^6−^ exhibits global aromaticity, as the template molecule signals appeared around 4 ppm, and THS signals showed significant differentiation due to shielding inside and deshielding outside the nanoring.

**Fig. 11:**
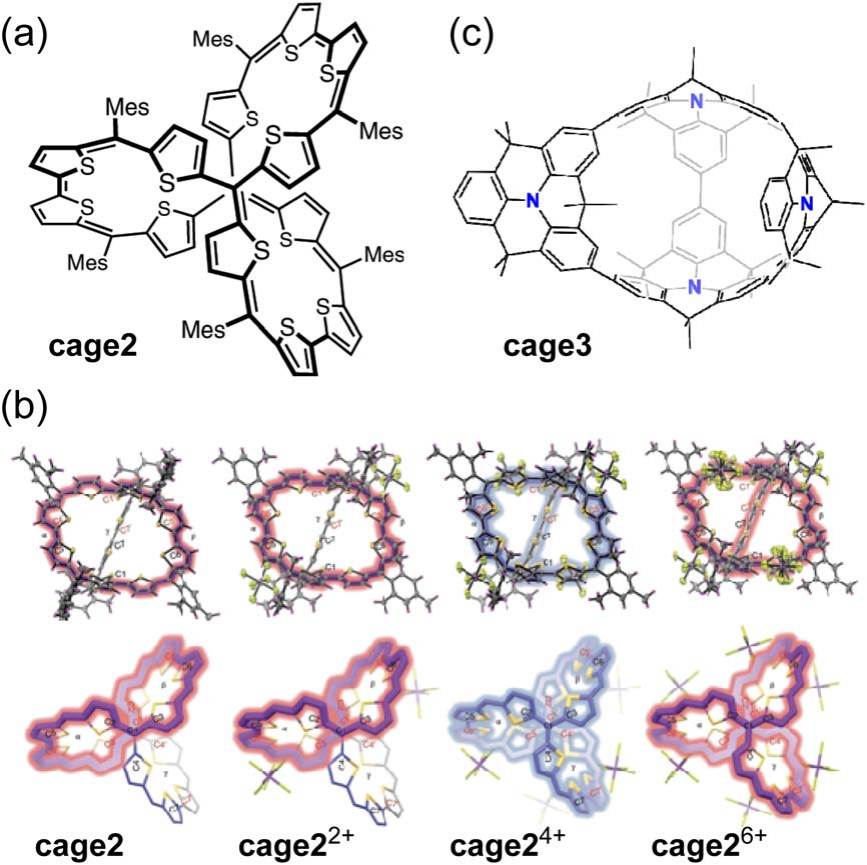
(a) Chemical structure of cage2. (b) X-ray structures of cage2, cage2^2+^, cage2^4+^, and cage2^6+^. Their dominant conjugation pathways are colored blue (antiaromaticity) and red (aromaticity). (c) Chemical structure of cage3. The color scheme for the crystal structures: H (magenta), C (grey), F (green), S (yellow), and Sb (purple).

Wu and co-workers reported a fully conjugated diradical molecular cage cage2 and its global aromaticity/antiaromaticity in various oxidation states (Fig. 11a).^117^ The diradicaloid cage, containing 12 conjugated thiophene units and 8 methine bonds, forms dication cage2^2+^, tetracation cage2^4+^, and hexacation cage2^6+^ upon chemical oxidation with 2, 4, and 6 equivalents of NO·SbF_6_, respectively (Fig. 11b). The neutral cage cage2 and its dication cage2^2+^ exhibit monocyclic conjugation pathways of 38π and 36π, satisfying the criteria for [4*n* + 2] Hückel aromaticity and [4*n*] Baird aromaticity, respectively. In contrast, the tetracation cage2^4+^ has 52 π-electrons delocalized throughout its 3D rigid framework, indicating 3D global antiaromaticity. The antiaromatic properties are further supported by ACID, NICS (NICS(0)_iso_ = +18.74 ppm), and 3D isochemical shielding surface (ICSS) calculations. ^1^H NMR measurements showed that the rotation of the bulky Mes group is slow on the NMR chemical shift timescale. The Mes protons pointing outward from the cage were highly shielded, whereas those pointing inward showed significant deshielding, with a large chemical shift difference (Δ*δ* = +6.85 ppm). Tetracation cage2^4+^ in CH_2_Cl_2_ exhibits a broad absorption band with *λ*_max_ at 628 and 933 nm, as well as a weak tail extending to 1700 nm. Hexacation cage2^6+^, with *D*_3_ symmetry and 50 globally delocalized π-electrons, exhibits [6*n* + 2] 3D global aromaticity. The demonstration of 3D global aromaticity/antiaromaticity is attributed to the rigid framework of the *D*_3_ symmetric tetracation cage2^4+^ and hexacation cage2^6+^.

The same group reported a fully π-conjugated open-cage molecule cage3 containing four dimethylmethylene-bridged triphenylamine units (Fig. 11c).^118^ The neutral cage3 can be oxidized by chemical oxidation with AgPF_6_ into dication cage3^2+^ or tetracation cage3^4+^ states, in which electrons are effectively delocalized over two or three dimensions. Neutral cage3 exhibits localized aromaticity in its individual benzene rings. Dication cage3^2+^ displays bicyclic (anti)aromaticity, with one macrocycle being aromatic (38π) and another macrocycle being antiaromatic (28π). Tetracation cage3^4+^ exhibits dominant 2D Hückel antiaromaticity in one of the macrocycles (36π).

## 4. Antiaromaticity in polymeric materials

**Fig. 12:**
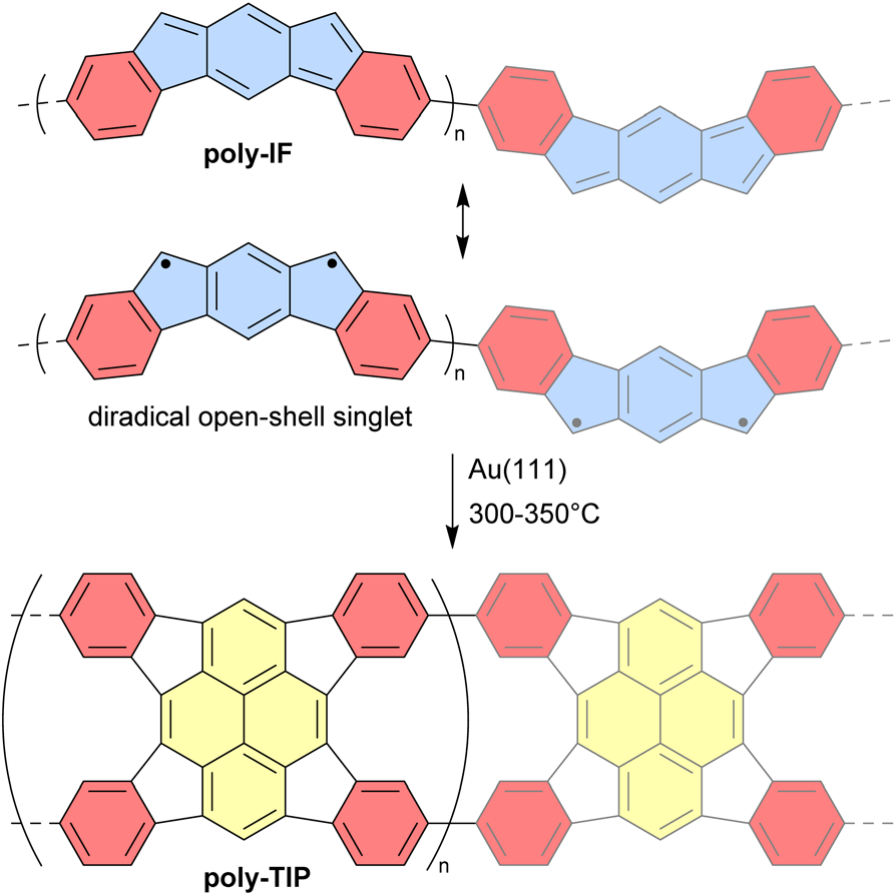
Resonance structures of poly-IF and its thermal conversion to poly-TIP. Different subunits are colored as follows: blue for antiaromatic *s*-indacene, red for aromatic benzene, and yellow for aromatic pyrene.

Unlike discrete assemblies, which can be isolated and finely characterized in the solution or solid state, polymeric materials are typically produced in bulk aggregates that may contain a significant number of defects that are difficult to identify. It is thus delicate to interpret their properties and link them to their expected idealized structure. For instance, it can be difficult to check the integrity of antiaromatic cores in polymeric materials based on antiaromatic building blocks. In some cases, however, the polymers may be generated or deposited on a surface as a fragment of a 1D strand or a 2D sheet that can then be locally analyzed in finer detail. In 2019, Di Giovannantonio *et al.* synthesized 1D conjugated polymers of antiaromatic indeno[2,1-*b*]fluorene poly-IF on a gold surface from *meta*-terphenyl derivatives (Fig. 12).^73^ Two strands of polymer poly-IF can fuse under the high temperature conditions required to form the indeno[2,1-*b*]fluorene core, creating 1D porous ribbons poly-TIP containing tetraindenopyrene monomers, thus leading to the coexistence of both polymers. The different structures were thoroughly characterized using STM and non-contact atomic force microscopy (nc-AFM). poly-IF possesses a very low band gap of 0.4 eV, whereas the band gap of poly-TIP is 2.2 eV, which is more common for organic semiconductors. Spin-polarized DFT calculations indicated that, out of the five possible indenofluorene isomers, indeno[2,1-*b*]fluorene possesses the second highest biradical character with an open-shell singlet ground state. The authors showed that poly-IF exhibits antiaromatic and radical characteristics similar to its isolated monomers. Such a conjugated open-shell polymer presenting multiple spins is a promising component for carbon-based spintronic circuits.

**Fig. 13:**
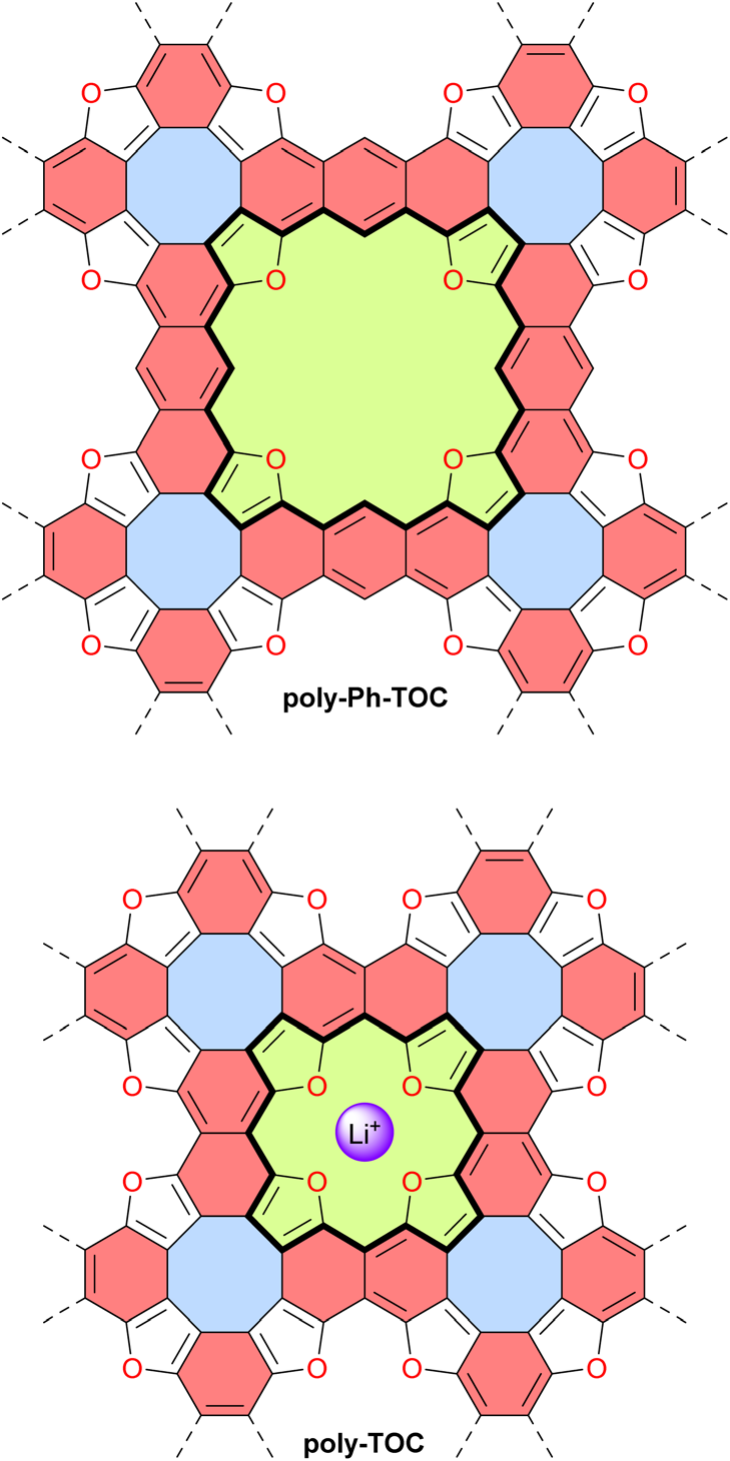
Tetraoxa[8]circulene-based 2D polymers poly-Ph-TOC and poly-TOC. The binding site of poly-TOC for Li^+^ is indicated. Different subunits are colored as follows: blue for antiaromatic cyclooctatetraene, red for aromatic anthracene or naphthalene, and green for potentially antiaromatic extended and normal tetraoxaisophlorin with 28 and 20 π-electron circuits indicated in bold.

In 2022, the group of Coskun reported the first tetraoxa[8]circulene-based porous 2D polymer poly-Ph-TOC in which the benzene units of the circulene are connected by another fused benzene ring forming an anthracene moiety (Fig. 13).^119^ This polymer exhibits semiconductor behavior and an unusual negative photoconductivity. Samples prepared at different temperatures display optical band gaps ranging from 1.3 to 1.9 eV which are relatively low values for organic semiconductors. Their electrical conductivity at room temperature ranges from 1 × 10^−6^ to 2 × 10^−5^ S cm^−1^ for undoped materials and reaches 4.1 × 10^−4^ S cm^−1^ upon doping with I_2_ vapor.

Very recently, the same group reported another related 2D polymer poly-TOC in which tetraoxa[8]circulenes are directly fused, forming naphthalene moieties (Fig. 13).^120^ The smaller pores of this polymer contain four converging oxygen atoms with a distal O⋯O distance similar to that of 12-crown-4 ether, a known selective receptor for lithium cations.^121^ Accordingly, the authors demonstrated that this polymer poly-TOC can selectively extract Li^+^ from aqueous solutions over other alkaline and alkaline-earth cations. The electronic and electrical properties of this polymer were not discussed in this study, but comparing poly-Ph-TOC and poly-TOC in these areas would be interesting for potential applications in organic electronics. Interestingly, the conjugated π-system around the pore macrocycle in these polymers (*i.e.*, central part of the structures in Fig. 13) contains 28 and 20 π-electrons for poly-Ph-TOC and poly-TOC, respectively. However, the antiaromaticity of these macrocycles was not discussed by the authors and should be verified by theoretical and/or experimental means.

**Fig. 14:**
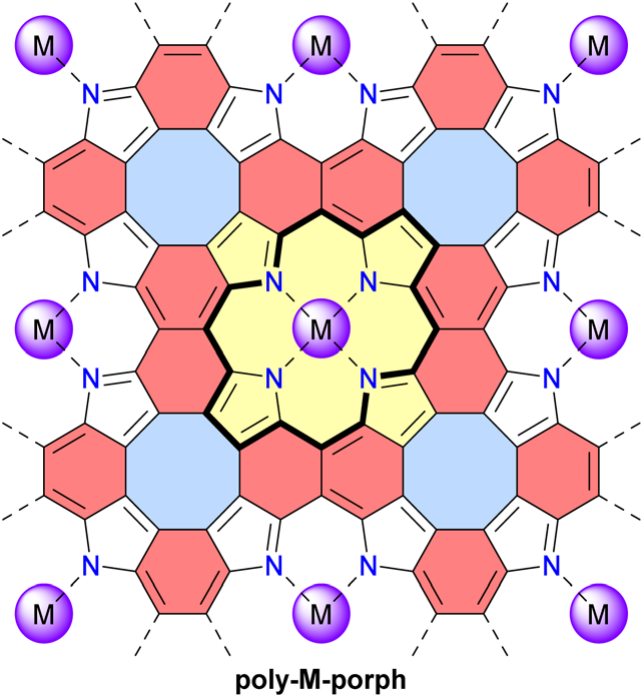
Structure of the metalloporphene (single-layered) and metalloporphite (multi-layered) 2D polymers poly-M-porph. Different subunits are colored as follows: blue for antiaromatic cyclooctatetraene, red for aromatic naphthalene, and yellow for aromatic porphyrin with the 18 π-electron circuit indicated in bold.

In 2023, the group of Michl reported the preparation and properties of metalloporphenes (single-layered) and metalloporphite (multi-layered) poly-M-porph, which are 2D polymers made of metalloporphyrin units bound at all β and *meso* positions (Fig. 14).^122^ Their structure contains antiaromatic planar cyclooctatetraene moieties and aromatic naphthalene moieties. This structure can also be viewed as a fused tetraaza[8]circulene polymer similar to poly-TOC presented above. However, the idealized structure of poly-M-porph contains fewer conjugated π-electrons since the aromatic porphyrin subunits (Fig. 14, 18 π-electrons) are oxidized compared to the corresponding antiaromatic tetraoxaisophlorin subunits in poly-TOC (Fig. 13, 20 π-electrons). Accordingly, it can reasonably be expected that the family of poly-M-porph polymers possesses a weaker antiaromatic character than poly-TOC. Samples of poly-Zn-porph porphite showed high electrical resistance at room temperature, whereas doping with I_2_ enabled electrical conductance, which is an expected semiconductor behavior but contrasts with the originally predicted metallic conductivity. In 2024, the same group further developed theoretical models of the electronic structure of these metalloporphenes to better understand the experimental results.^123^ Their models revealed that poly-Zn-porph possesses an electronic structure analogous to that of antiaromatic molecules, making it susceptible to a Peierls distortion sufficiently large to explain its semiconductivity. In parallel, these models predicted that other metalloporphenes should exhibit a variety of electrical conductivity behaviors, ranging from diamagnetic and paramagnetic semiconductors to metallic conductors.

**Fig. 15:**
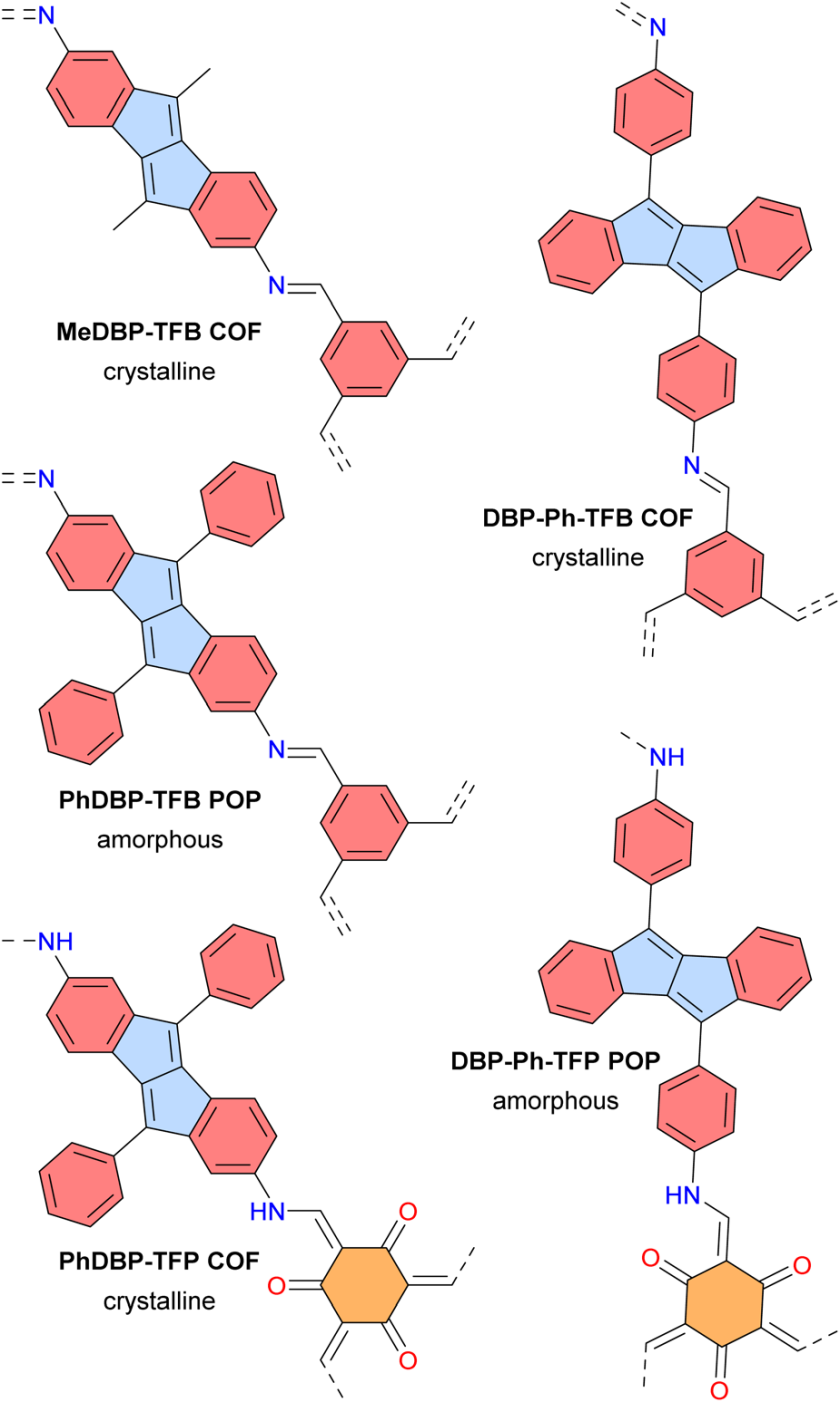
Dibenzopentalene-based COFs and POPs. Different subunits are colored as follows: blue for antiaromatic pentalene, red for aromatic benzene, and orange for tris-β-ketoenamine–cyclohexane.

In 2023, the groups of Dumele and Esser reported crystalline porous covalent organic frameworks (COFs) and amorphous porous organic polymers (POPs) based on antiaromatic dibenzopentalene building blocks (Fig. 15).^124^ These materials display broad optical absorption bands extending up to 800 nm and positive photoconductivity. The PhDBP-TFP COF is a semiconductor with a low conductivity of 1 × 10^−12^ S cm^−1^ at room temperature, which increased to 4 × 10^−8^ S cm^−1^ upon doping with I_2_ vapor. Li-organic batteries using the crystalline PhDBP-TFP COF as a positive electrode exhibited a capacity of 26 mA h g^−1^, whereas the amorphous DBP-Ph-TFP POP showed a lower capacity of 14 mA h g^−1^, both at a high potential of 3.95 V *vs.* Li/Li^+^. Furthermore, the battery containing the PhDBP-TFP COF outperformed an analogous aromatic COF containing biphenyl moieties instead of dibenzopentalene. These good results for the antiaromatic materials are attributed to localized redox activity at the antiaromatic dibenzopentalene moieties. Indeed, antiaromatic compounds tend to be stabilized in their oxidized and reduced states.

**Fig. 16:**
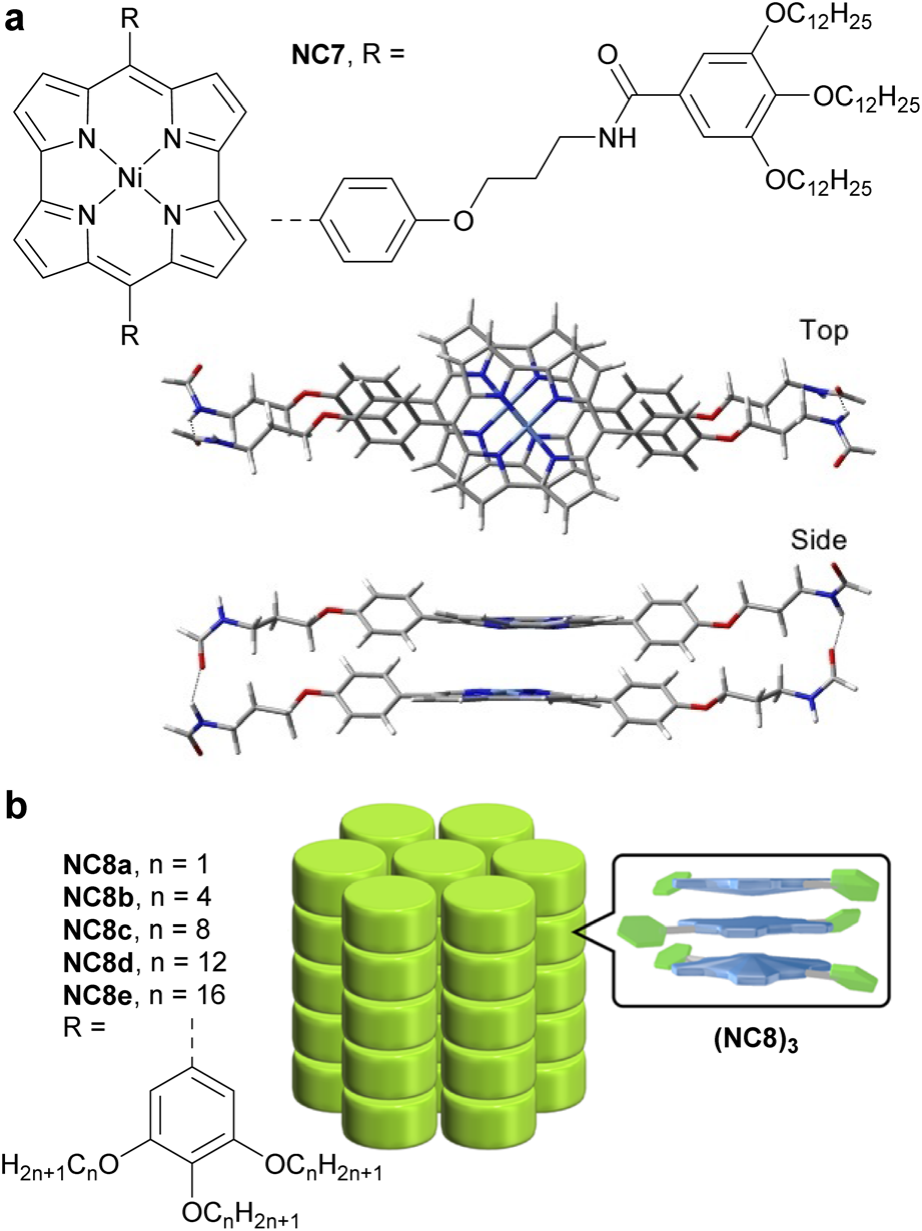
(a) Structure of amide-decorated Ni-norcorrole NC7 and its DFT-optimized slipped stacking assembly. The color scheme for the crystal structures: H (white), C (grey), N (blue), O (red), and Ni (cyan). (b) Structure of the Ni-norcorrole family NC8 and the assembly of trimers (NC8)_3_ into a columnar superstructure.

In 2022, Ukai *et al.* reported a supramolecular polymer made of stacked antiaromatic Ni-norcorroles (NC7)_*n*_ (Fig. 16a).^125^ The assembly process relies on π–π stacking and hydrogen bonding between amide groups on the norcorrole's side chains, resulting in well-aligned norcorrole cores. Unlike the covalent materials described above, this supramolecular polymer does not possess an extended π-conjugated system; however it exhibits charge-transporting ability in the form of photoconductivity, due to the close face-to-face stacking and good alignment of the norcorrole units, which create electronically conductive pathways. In solution at 298 K, the sum of the isotropic electron and hole mobility is ∑*μ* = 1 × 10^−3^ cm^2^ V^−1^ s^−1^, which is three times higher than that of an aromatic Zn-porphyrin stacked supramolecular polymer. In the solid state, NC7 exhibits a photoconductivity similar to that of *meso*-dimethyl-Ni-norcorrole.^126^

In 2024, Ishikawa *et al.* studied a family of Ni-norcorroles NC8 decorated with aliphatic chains of various lengths (Fig. 16b).^127^ These norcorroles initially form self-assembled π-stacked trimers (NC8)_3_, which further form various assemblies in the single crystal and thermotropic liquid crystal states, depending on the aliphatic chain length. Long aliphatic chains have proven to be key structural elements for forming mobile columnar arrangements and liquid crystal phases. NC8d with dodecyl chains in its liquid crystal state showed the highest photoconductivity (5.2 × 10^−8^ m^2^ V^−1^ s^−1^) among the NC8 derivatives, which is rationalized by the closer stacking and better alignment of NC8d units, creating a more effective electric conductive pathway.

## 5. Conclusion and future outlook

Who could have imagined that at the time when Hückel defined aromaticity and antiaromaticity, antiaromatic molecules would become building blocks for functional assemblies? Over the past two decades, the advent of innovative molecular design and synthetic methods has circumvented the significant instability of antiaromatic molecules, their main limitation, making them manipulable under ambient conditions. In addition, the use of appropriate chemical bonds and non-covalent interactions has allowed the construction of intriguing superstructures that benefit from antiaromatic components and, in some cases, exhibit enhanced antiaromatic-specific behaviors emerging from the assembly. As a result of these achievements, the specific properties of antiaromatic molecules (*e.g.*, paratropic currents, magnetic deshielding effects, and face-to-face orbital interactions) are gradually becoming better understood. In particular, it has been demonstrated that assemblies of antiaromatic molecules display distinct chemical and physical properties compared to their aromatic counterparts, such as superior electric conductivities, strengthening the magnetic deshielding space of separate antiaromatic building blocks, and the mutual stabilization of stacked antiaromatic rings *via* the emergence of 3D aromaticity. These findings are expected to pave the way for new research directions in the fields of organic chemistry and supramolecular chemistry.

Discrete antiaromatic-based assemblies are elegant objects that have gained increasing attention in the scientific literature in recent years. They showcase the mastery of chemists over matter by controlling the infamously unstable antiaromatic rings; however, their practical use remains elusive as most studies are focused on fundamental aspects. There are some proposed applications, such as using antiaromatic-based receptors as NMR chemical shift reagents to displace the signals of guest compounds downfield, but this application is underwhelming compared to the significant investment in preparing antiaromatic-based assemblies. Future efforts should be directed toward developing applications with broader societal impact, such as finding unusual catalytic properties for these systems and leveraging their specific redox and/or photophysical properties. Indeed, discrete assemblies frequently possess cavities that can encapsulate substrates. The resulting host–guest complexes ensure close proximity between the substrate and the antiaromatic rings over a sufficiently long timeframe, thus promoting their interaction. Such behavior has been utilized previously to promote catalysis in cages that are not based on antiaromatic rings.^128^ The small HOMO–LUMO gap, characteristic of antiaromatic rings, may allow for the efficient harvesting of low-energy photons (*e.g.*, in the red to infrared spectrum) to drive photocatalytic reactions on guest substrates. Redox and photoredox reactions could benefit from the high HOMO and low LUMO levels of antiaromatic rings, which allow them to donate and accept electrons, respectively. Furthermore, antiaromatic rings tend to be stabilized in their oxidized dicationic or reduced dianionic states, which are aromatic. This stabilization should prevent the reverse redox reaction; however it may introduce new challenges in identifying suitable sacrificial electron donors or acceptors for closing the catalytic loop. The diradical character of antiaromatic rings could perhaps be used to promote radical processes within the cavity of antiaromatic-based discrete assemblies. However, this approach appears intricate to develop since antiaromatic building blocks are likely to react as radical species and degrade. On a more fundamental level, the 3D aromaticity stabilization of closely π-staked antiaromatic rings could be utilized to stabilize particularly unstable antiaromatic species residing as guests within the cavity of antiaromatic-based assemblies, thereby facilitating their characterization and detailed study. It should be noted that these potential applications are highly speculative since such uses of antiaromatic rings, whether isolated or assembled, have yet to be demonstrated. The Ni-norcorrole assembly cage1 that we previously reported could serve as an entry point for exploring these avenues, as it is the first and, to this day, only antiaromatic-based assembly featuring a cavity that is completely isolated from the external environment and fully enclosed by antiaromatic walls.^103^

In contrast, polymeric materials based on antiaromatic systems, although less reported, have demonstrated clear potential, particularly in organic electronics, thanks to the various specific properties of antiaromatic rings, such as small HOMO–LUMO gaps and high carrier mobility. The limited number of reports is likely due to the relative instability of antiaromatic rings, which restricts synthetic routes to polymerize them while retaining their integrity. Accordingly, all reported antiaromatic-based covalent polymeric materials have been made from antiaromatic/aromatic fused rings, such as tetraoxa[8]circulene, to alleviate the instability of antiaromatic rings with stable aromatic ones. Despite this compromise, the durability of such materials remains unknown and must be ascertained before moving forward with functional applications.

To the best of our knowledge, there are only five patents (excluding a duplicate) that cover the use of antiaromatic-based molecules or materials, which are focused on applications in organic electronics, particularly luminescence.^129–133^ These patents were all published within the last 10 years, indicating recent yet discreet interest in the industrial applications of antiaromatic compounds owing to their unique electronic properties.

We expect that the library of antiaromatic compounds and assemblies will continue to grow in the coming years, pushing beyond the limits of their current aromatic counterparts. We believe that antiaromatic heteroisophlorins are particularly promising building blocks owing to their strong antiaromatic character and structural similarity to porphyrins, which are broadly used as components in cage compounds and polymeric frameworks. Accordingly, we wish to see further developments in their synthesis and assembly into larger architectures.

We hope that this perspective can serve as an entry point for newcomers to the field of antiaromatic-based assemblies and promote further developments in this exciting area.

## Author contributions

R. L. and M. Y. performed the literature search and wrote the manuscript.

## Conflicts of interest

There are no conflicts to declare.

## Data Availability

All new data on which this publication is based are presented in the main text.
